# Habitat conditions filter stronger for functional traits than for phenology in herbaceous species

**DOI:** 10.1002/ece3.11505

**Published:** 2024-06-04

**Authors:** Till J. Deilmann, Josephine Ulrich, Christine Römermann

**Affiliations:** ^1^ Institute of Ecology and Evolution Friedrich‐Schiller‐University Jena Jena Germany; ^2^ Senckenberg Institute for Plant Form and Function Jena Jena Germany; ^3^ German Centre for Integrative Biodiversity Research (iDiv) Halle‐Jena‐Leipzig Leipzig Germany

**Keywords:** botanical garden, first flowering day, grassland, habitat conditions, phenology, plant functional traits

## Abstract

An increasing number of studies in botanical gardens are investigating species' responses to climate change. However, the influence of local environmental or habitat conditions such as soil nutrient status or microclimate on phenology and the link between morpho‐physiological functional traits and phenological stages are poorly understood, making it difficult to extrapolate patterns from botanical gardens to natural environments. Therefore, we selected herbaceous species growing in two semi‐natural habitats, namely, semi‐dry grasslands (SDGs) and mesophilic grasslands (MGs) and the botanical garden of Jena (Germany) to investigate the influence of habitat conditions on interspecific and intraspecific patterns in phenology, functional traits and their associations. For 16 species, we monitored leaf and flowering phenology weekly for 133 populations from the three habitats, measured morpho‐physiological traits (i.e., whole plant, leaf and reproductive traits), as well as habitat conditions and compared the measurements across habitats. Multivariate analyses revealed that morpho‐physiological traits conspicuously showed stronger differences between habitats compared to phenological traits. Populations on MG showed temporal niche segregation, whereas populations on SDG showed flowering synchrony. Boosted Regression Trees showed that morpho‐physiological traits, especially reproductive traits, strongly influenced phenological traits and that the trait‐phenology relationships were highly habitat‐specific. We conclude that species phenology is broadly similar between botanical gardens and local habitats. However, phenological responses to the environment may be constrained by a certain suite of correlated traits due to ecological plant strategies that vary across habitats. The effect of habitat conditions on morpho‐physiological functional traits and phenology‐trait relationships is important and should not be neglected at local scales, implying consequences at larger scales.

## Graphical abstract

For 16 species, we monitored leaf and flowering phenology weekly for 133 populations in the botanical garden of Jena and semi‐dry and mesophilic grasslands and compared them across habitats for whole plant, leaf and flower traits, across and phenological stages. Multivariate analyses showed that habitat conditions filtered stronger for functional traits than for phenology and populations on MG showed temporal niche segregation, whereas populations on SDG showed flowering synchrony. Our results show that species phenology was broadly similar between botanical gardens and local habitats but that for functional trait and for trait‐phenology associations, habitat should not be neglected.

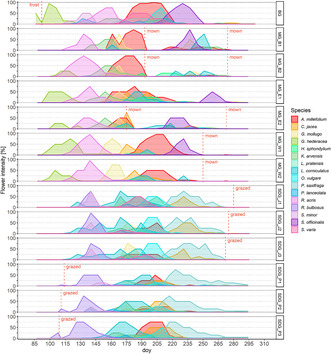

## 1 INTRODUCTION

Plant phenology is generally defined as the study of recurring life development stages in plants and its study has a long history which can be dated back thousands of years (Piao et al., 2019). In the beginning, mainly used for agricultural purposes, phenological observation has evolved into a scientific interest investigating the timing of biological events. Especially during the last decades facing global change, it has gained continuously more attention and has become a comprehensive and complex field of research with the main aim of understanding the responses of biological communities to a changing environment (Parmesan & Yohe, 2003; Piao et al., 2019) and it has also been defined as the “fingerprint of climate change” (Root et al., 2003). Phenology is generally considered to be a plant functional trait (Violle et al., 2007). In this study, however, we distinguish between phenological stages and other functional traits in a way that the former represents traits being linked to the time development of plants while the latter represents morpho‐physiological traits.

Many studies have already shown an impact of warming climate on the phenology of trees, shrubs and crops, overall resulting in a prolonged growing season (e.g., Chmielewski & Rötzer, 2001; König et al., 2018; Menzel & Fabian, 1999; Vitasse et al., 2011). However, since 85% of the plants in temperate ecosystems are estimated to be non‐woody (Ellenberg & Leuschner, 2010), research in herbaceous species is crucial to understand ecosystem responses to climate change. Studies have already found that the progressively changing climate led to a prolonged growing season in non‐woody plants resulting from earlier springs, delayed autumns and longer periods without frost (Dunnell & Travers, 2011; Matsumoto et al., 2003; Menzel & Fabian, 1999). Many studies reported changes in major spring phenological stages, such as shifts in leaf out and first flowering day (Bucher et al., 2018; Dunnell & Travers, 2011; Fitter & Fitter, 2002; König et al., 2018; Menzel et al., 2006; Peñuelas et al., 2004). The direction of change, however, was often reported to depend on both species and phenological stages investigated. Yet, general trends were found. For instance, the first flowering day mainly showed a negative relationship with increasing temperature (Bock et al., 2014; Bucher & Römermann, 2020; König et al., 2018) but a more ambiguous relationship with soil moisture (Nord & Lynch, 2009).

Changes in autumn phenology, which refers to events at the later stages of plants' life cycles such as fruiting or leaf senescence, are less intensively studied despite being ecologically and evolutionarily important (Gallinat et al., 2015; Richardson et al., 2013, but see Bucher & Römermann, 2021; Menzel et al., 2006; Peñuelas et al., 2004). For instance, leaf senescence, indicating the end of the growing season, affects the carbon and nutrient allocation of the plant in the current and subsequent years, making its timing crucial (Estiarte & Penuelas, 2015). Similar to spring events, autumn phenology was also shown to be impacted by changing climatic conditions (Bucher & Römermann, 2021; Walther et al., 2002) with studies mainly reporting delayed leaf senescence (Bucher & Römermann, 2021; Estiarte & Penuelas, 2015; Ibáñez et al., 2010). To get a comprehensive picture of the phenological responses of species, it is necessary to investigate their whole life cycle including both spring and autumn phenological stages in further studies.

In recent years, increasingly more studies on phenology have taken place in botanical gardens (Harper et al., 2004; Nordt et al., 2021; Panchen et al., 2015; Primack et al., 2021). Botanical gardens provide a perfect setting for phenological research as they harbour numerous plant species from various habitats and climates and offer the possibility to monitor the full life cycles of plants together with their trait composition (Nordt et al., 2021). Their global distribution and diversity of taxa make them ideal for use as common garden experiments over large spatial and temporal scales (Primack et al., 2021). Botanical garden studies in seasonal regions also found a general trend of advanced spring events and delayed autumn events with increasing temperatures (Harper et al., 2004; Nordt et al., 2021; Sparks et al., 2011; Sporbert et al., 2022).

Besides the large‐scale effects of global warming, also local habitat conditions affect the phenology of plant species (e.g., Plos et al., 2024). These responses can be investigated in different habitats, which vary in habitat conditions. Distinct habitat conditions lead to locally adapted plant populations varying in morpho‐physiological plant traits and phenology (Clausen et al., 1940; Joshi et al., 2001; Linhart & Grant, 1996; Reisch & Poschlod, 2011). For instance, species in dry compared to mesophilic environments typically show higher leaf dry matter content (LDMC) and have thicker leaves, while their values for leaf nitrogen concentrations, leaf area and specific leaf area (SLA) are typically low (Blumenthal et al., 2020; Cornwell & Ackerly, 2009). Therefore, habitat conditions filter for species that exhibit specific traits which are needed to survive in the respective environment (Keddy, 1992; Le Bagousse‐Pinguet et al., 2017). Consequently, the species composition and hence the functional traits and phenological stages of the species of the respective community are shaped by the environmental conditions of the habitat. Across habitats, focussing on the same species set helps to get the real influence of the habitat‐specific changes and consequently enables us to assess to which extent data from, e.g., botanical gardens overlap with data from natural habitats.

In addition to habitat conditions, several studies have shown that plant traits mediate phenological responses to changes in climate (Bolmgren & Cowan, 2008; Bucher et al., 2018; König et al., 2018; Sporbert et al., 2022). Vegetative traits, such as plant height, SLA or LDMC and generative traits, such as flowering time or pollination type, reflect evolutionary adaptations to local habitat conditions (König et al., 2018; Pérez‐Harguindeguy et al., 2016). Hence, functional traits are directly affected by changing habitat and climatic conditions (Lang et al., 2020; Reisch & Poschlod, 2011) and, therefore, can function as predictors of species responses to changing conditions. For example, Bucher et al. (2018) and Bucher and Römermann (2021) showed in a study along elevational gradients that fast‐growing species, represented by a high SLA, flowered and senesced earlier while species with a high LDMC senesced later. Results from botanical garden studies showed that leaf area and canopy height are among the most important traits driving variations in vegetative and generative phenology, that is, taller plants and large‐leaved species showed a later time of initial growth and flowered, fruited and underwent leaf senescence later (Sporbert et al., 2022).

These studies show that the association of phenological stages and other functional traits are important to understand different life history strategies among species. Phenological responses may be constrained by a set of correlated traits based on the ecological strategy which is exhibited in a certain habitat. To be able to fully understand species‐ and habitat‐specific phenological variation, we therefore need to integrate a comprehensive understanding of the other functional traits that are present in the plant community.

Since there is a lack of knowledge of how these relationships work across habitats, we aimed to investigate whether botanical garden patterns of phenology and other functional traits, as well as their association, are broadly representative of those in natural populations. Following on from this, we here examine how important the effect of local habitat conditions is on the phenology, on the morpho‐physiological traits and their associations. To our knowledge, there has been no study that directly compared functional traits, phenological stages, or their association between a botanical garden and the species' natural habitats. To address this knowledge gap, we observed the phenological stages and morpho‐physiological traits for a total of 133 populations of 16 herbaceous species in the botanical garden of Jena (Thuringia, Germany) and in six semi‐dry and six mesophilic grasslands under comparable climatic conditions in the same region over an entire growing season. More specifically, we aimed to answer the following questions:

1. How do morpho‐physiological functional traits differ between semi‐dry and mesophilic grasslands and the Jena botanical garden?
2. Are there differences in the timing of the phenological stages between these habitats?
3. Are trait‐phenology relationships consistent across habitats or do they depend on habitat conditions?

Answering these questions will provide deeper insights into the interactions between phenology and plant traits under various habitat conditions and will help determine to which extent results from observations in botanical gardens can be transferred to semi‐naturals habitats.

## 2 MATERIALS AND METHODS

### 2.1 Study area and experimental setup

**FIGURE A1:**
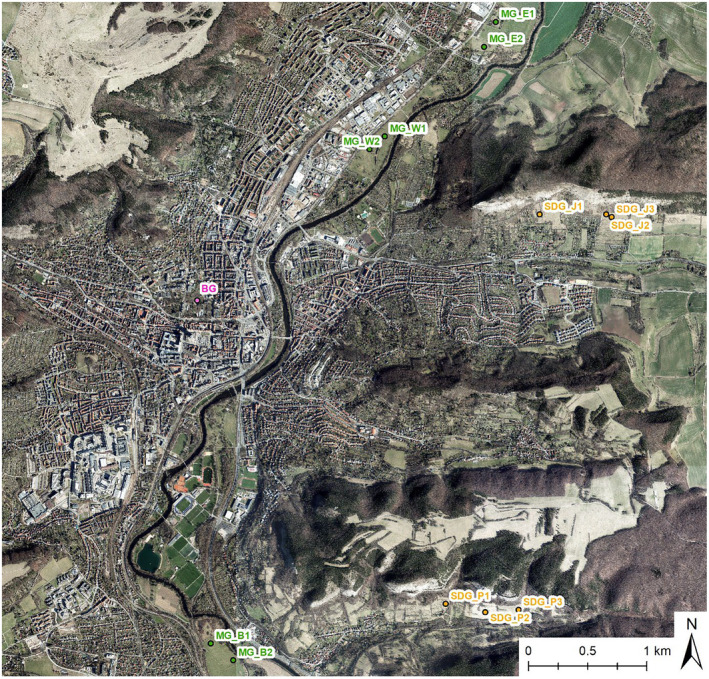
An overview map of the distribution of the study sites in and around Jena, Thuringia (Central Germany). BG, botanical garden (pink); MG, mesophilic grassland (green); SDG, semi‐dry grassland (yellow). Orthophotos were retrieved from https://www.geoportal‐th.de/de‐de/ (Access: May 2020).

As study area, we chose the botanical garden in Jena (BG), Thuringia (Germany) and two semi‐natural habitat types ‘mesophilic grasslands’ (MG) and ‘semi‐dry grasslands’ (SDG) around Jena because they differ strongly in their habitat conditions and are rich in herbaceous species. For each semi‐natural habitat, we selected six grassland sites, making a total of 13 different sites and three habitats (cf. Appendix Figure A1). MG is typically mown twice, and SDG is typically grazed once, but due to changes in tenure, but also due to adaptations of the management to weather conditions, management has not been completely consistent across sites and previous years (see Table 1). In this setting it was not possible to include more than one botanical garden, therewith this is only represented with *N* = 1. However, here we assume that this study design comparing patterns from a botanical garden with the patterns in semi‐natural habitats is representative of other botanical garden – semi‐natural grassland comparisons within similar climate conditions of a region.

**TABLE 1:** Overview of the study sites of the botanical garden (BG), the mesophilic grasslands (MG) and the semi‐natural dry grasslands (SDG) regarding the number (#) of observed target species and the number (#) of managements per site.

Habitat	Site abbreviation	Site full name	# observed target species	# managements
BG	BG_*species*	BG_*species*	13	0
MG	MG_B1	Burgaupark 1	9	2
MG	MG_B2	Burgaupark 2	8	2
MG	MG_E1	Jena Experiment 1	11	0
MG	MG_E2	Jena Experiment 2	10	2
MG	MG_W1	Wiesenstraße 1	10	1
MG	MG_W2	Wiesenstraße 2	9	1
SDG	SDG_J1	Jenzig 1	11	1
SDG	SDG_J2	Jenzig 2	11	1
SDG	SDG_J3	Jenzig 3	11	1
SDG	SDG_P1	Pennickental 1	10	1
SDG	SDG_P2	Pennickental 2	10	1
SDG	SDG_P3	Pennickental 3	10	1

*Note*: The number of managements refers to the mowing events on MG and grazing events on SDG. Information on habitat conditions can be extracted from Table A1. A map showing the location of the different sites can be found in the Appendix Figure A1.

In each of the five replicate mesophilic grasslands and each of the five replicate semi‐dry grasslands (Table 1, Figure A1), a 20 m × 20 m observational area was established, within which five replicate plots of 2 m × 2 m were set up each. Within the 20 m × 20 m area, we monitored phenology weekly and measured plant functional traits once for all selected species. In the BG, these observations and measurements took place in the species beds. The species beds covered approximately 1 m^2^ for all selected species (cf. Nordt et al., 2021). As *Heracleum sphondylium* was only present in a wild lawn in BG, we set up a 2 m × 2 m plot to include at least five individuals to capture variation in phenological stages and other functional traits as was done for the other species. Within the 2 m × 2 m plots, we monthly measured soil moisture and leaf area index (LAI) and performed vegetation surveys (see below). In the BG, these observations and measurements took place in the species beds.

### 2.2 Species selection

We selected 16 species in total based on their characteristic occurrence in MG and SDG according to Ellenberg and Leuschner (2010) and expert knowledge. All selected species were also present in BG.

The species were chosen to cover several families (9) and genera (14) and to belong to the growth form of hemicryptophytes as these strongly dominate these habitats (see Table 2). Taxonomy follows Euro + Med PlantBase (2006).

**TABLE 2:** All observed species in the study, separated by habitat mesophilic grassland (MG) and semi‐dry grassland (SDG) that were also monitored in the Botanical Garden Jena.

Mesophilious grassland			Semi‐dry grassland		
Species	Family	No.	Species	Family	No.
*Achillea millefolium* agg.	Asteraceae	6	*Achillea millefolium* agg.	Asteraceae	6
*Centaurea jacea* L.	Asteraceae	4	*Centaurea jacea* L.	Asteraceae	6
*Galium mollugo* agg.	Rubiaceae	6	*Galium mollugo* agg.	Rubiaceae	6
*Knautia arvensis* (L.) DC.	Caprifoliaceae	4	*Knautia arvensis* (L.) DC.	Caprifoliaceae	6
*Lotus corniculatus* L.	Fabaceae	6*	*Lotus corniculatus* L.	Fabaceae	6*
*Plantago lanceolata* L.	Plantaginaceae	5	*Plantago lanceolata* L.	Plantaginaceae	6
*Glechoma hederacea* L.	Lamiaceae	6	*Origanum vulgare* L.	Lamiaceae	6
*Heracleum sphondylium* L.	Apiaceae	5	*Pimpinella saxifraga* L.	Apiaceae	6*
*Lathyrus pratensis* L.	Fabaceae	5	*Securigera varia* (L.) lassen	Fabaceae	3
*Ranunculus acris* L.	Ranunculaceae	6	*Ranunculus bulbosus* L.	Ranunculaceae	6*
*Sanguisorba officinalis* L.	Rosaceae	4	*Sanguisorba minor* scop.	Rosaceae	6

*Note*: Cells highlighted in grey indicate match species occurring in all three habitats. No. = number of sites where the species were observed, and an asterisk * indicates species that were not present in the botanical garden but were included to compare the two habitats.

The analyses below focus on two sets of species as not all species were present at every site. The first set of species includes all 16 species, the second set of species includes only those six species occurring in all three habitats (in the following referred to as ‘match species’; Table 2). The analyses based on the first species set nicely unravel the patterns in traits and phenology of the most dominant and characteristic species between grassland types (in total 11 species per habitat) and assess the impact of habitat filters in a general way. The second set of species, consisting of match species, accounts for the effect of intraspecific variation more comprehensively.

### 2.3 Habitat conditions

All sites were characterised concerning air temperature and relative humidity, aspect, inclination, soil depth and soil nutrients:

We installed one weather station (HOBO data logger U23 Pro v2; Onset Computer Corporation, USA) with a solar radiation shield at each site at a standardised height of 1.5 m to collect data on air temperature (°C) and relative air humidity (%) in hourly intervals. Due to the high variation in sites covered in BG, four data loggers were established, and the data of the closest one for each species' bed was used for further analyses. Due to technical problems, missing values were predicted from linear models from other available data as outlined in Appendix Note A1.

All study sites were characterised concerning aspect (°) using a compass and inclination (°) using the laser rangefinder TruPulse 200 (Laser Technology Inc, USA). We measured soil depth (cm) with a soil depth stick three to five times at every plot, depending on the variation.

Soil nutrients were analysed for one mixed soil sample per plot (*N* = 5 per site), consisting of three sub‐samples of the main rooting horizon of the species, i.e., the upper 20 cm of soil. All soil samples were taken in April 2020. In the BG, the procedure described above was conducted for each species bed and was done before the plots were fertilised. The soil was dried at 60°C for 1 week. Afterwards, all samples were sieved to 1 mm, ground finely in a mortar and stored in a dry place until further analyses.

To investigate the pH and electric conductivity (EC; μS/cm) of the soil, we followed the protocol of soil analysis for mineral soils of the Institute of Geography, FSU Jena (AG Soil Science, 2020). Soil carbon, nitrogen and sulphur content (%) were determined by high‐temperature combustion with the organic elemental analyser vario MAX cube (Elementar Analysensysteme, Germany) in the laboratory of the Institute of Geography at the Friedrich Schiller University Jena.

Soil moisture (%vol) was taken once a month following three preceding days without rain. Per plot, five measurements were conducted using the HH2 Moisture Meter and ML3 ThetaProbe (Delta‐T Devices, UK). Similarly, we monthly measured the leaf area index (LAI) with the LAI‐2200 Plant Canopy Analyzer (Li‐Cor Inc, USA).

To characterise species composition, we conducted two vegetation surveys for each plot on each site, one in early June before the first cut on the MG and the grazing treatment in SDG and one end of August/early September after the management treatments. To estimate species abundance, we used the Schmidt scale, which is a fine‐scaled tool for vegetation surveys (from Pfadenhauer, 1997) using the following classes of cover: 0, 1, 5, 15, 25, 50, 75, 95 and 100%. To characterise the habitat based on occurring vegetation, we calculated community‐weighted mean Ellenberg indicator values (EIV; Ellenberg & Leuschner, 2010). The EIVs assign certain values to plant species, thus describing the habitat conditions by the occurring species composition in terms of nutrients (N), light conditions (L), temperature (T), soil moisture (F), soil reaction (R) and continentality (K).

### 2.4 Plant trait measurements

At peak flowering, we measured plant traits following standardised protocols (reproductive traits: Kearns & Inouye, 1993, plant traits: Cornelissen et al., 2003; Pérez‐Harguindeguy et al., 2016). An overview of the measured traits, units and their ecological relevance is summarised in Table 3. Per site and species, five healthy individuals were sampled if available.

**TABLE 3:** Overview of measured plant traits, including whole plant‐, leaf‐, and floral traits and their respective ecological function.

Plant trait	Unit	Ecological function	References
Specific leaf area (SLA)	mm^2^/mg	Productivity, competitive ability	Pérez‐Harguindeguy et al. (2016), Garnier (1992)
Leaf nitrogen content	%	Productivity, competitive ability	Pérez‐Harguindeguy et al. (2016)
Leaf carbon content	%	Resistance, leaf lifespan	Pérez‐Harguindeguy et al. (2016)
Leaf dry matter content (LDMC)	mg/g	Resistance, leaf lifespan	Pérez‐Harguindeguy et al. (2016), Blumenthal et al. (2020)
Leaf thickness	mm	Resistance, leaf lifespan	Pérez‐Harguindeguy et al. (2016), Blumenthal et al. (2020)
Vegetative height	cm	Competitive ability	Pérez‐Harguindeguy et al. (2016), Gaudet and Keddy (1988), Falster and Westoby (2003)
Generative height	cm	Dispersal, fecundity	Thomson et al. (2011), Vile et al. (2006)
Plant width	cm	Competitive ability	Gaudet and Keddy (1988)
Nectar sucrose content	%	Pollinator reward	Fornoff et al. (2017)
Flower size	cm	Reproductive success, pollination	Hegland and Totland (2005), Fornoff et al. (2017)
Flower density	m^−2^	Reproductive success, pollination	Sih and Baltus (1987), Comba (1999), Hegland and Totland (2005)

In situ measurements contained plant width, vegetative and generative height, individual flower number, as well as flower density and size. The flower size represents the ‘longest flower dimension’ to make radial symmetric and zygomorphic flowers comparable.

To measure leaf traits, two healthy leaves were taken per individual, if available, one basal and one stem leaf, wrapped in moist paper tissue and stored in a zip‐lock plastic bag in the fridge until measurement within a maximum of 3 days. Per leaf, we recorded leaf thickness with a digital calliper gauge at the middle of the lamina, LDMC as the ratio of dry mass to fresh mass and SLA by dividing the one‐sided fresh leaf area by its dry mass. We obtained leaf area by calculating the actual area from scans using the *LeafTraits* R‐package (M. Bernhardt‐Römermann, unpublished).

For analyses of leaf nitrogen and carbon content, we assessed nutrients using high‐temperature combustion with the CHNS elemental analyser vario EL cube (Elementar Analysensysteme, Germany) at the laboratory of geobotany of Martin‐Luther University Halle (Germany).

### 2.5 Phenological monitoring

For each species on each site (20 m × 20 m), we recorded the flowering and senescence intensity as well as the presence of ripe fruits every week on a population level following the *PhenObs* protocol (Nordt et al., 2021). Flowering and senescence intensity were estimated in the following classes: 0, 1, 5, 15, 25, 50, 75, 95 and 100%. From this data, we extracted the first flowering day (FFD), the maximum flowering intensity (FI_max_) and the flowering duration (FD) as a count of days where functional flowers (= exposed anthers of inflorescences) were recorded in a population. To avoid potential confounding factors, we chose to evaluate FD with at least 5% flowering only (FD_5_).

In addition, we recorded autumn phenology, which is defined here to include leaf senescence (LS) and fruiting parameters, although these can also happen earlier in the year. We chose two different stages of LS: the onset of LS which was determined as the day when 5% of the leaves of a population showed senescence (LS_5_) and the peak of LS defined as the day when 50% of the leaves of a population displayed senescence (LS_50_) following Bucher and Römermann (2021). Not every population reached LS_5_ or LS_50_ during the period of observation which automatically excluded them from corresponding analyses. Furthermore, we determined the day of the first ripe fruit (FRF) of a population.

*Galium mollugo* in BG had to be excluded from analyses involving flowering and fruiting stages due to early pruning.

### 2.6 Data analysis

All analyses were conducted in R version 4.2.3 (R Core Team, 2023) using the packages *dplyr* (Wickham et al., 2023), *reshape2* (Wickham, 2007) and *openxlsx* (Schauberger & Walker, 2023) for data management and *ggplot2* (Wickham, 2016) for visualisation purposes. Other packages for analyses are mentioned directly in the text.

For descriptive analyses, we tested for differences in habitat conditions between habitats, using an ANOVA or a Kruskal–Wallis test, respectively, depending on the distribution of the data and corresponding post‐hoc tests (pairwise *t*‐test and a pairwise Wilcoxon‐rank sum test with Holm–Bonferroni correction for multiple tests, respectively). To characterise the different habitats in terms of vegetation composition, we conducted a detrended correspondence analysis (DCA) as the length of the first axis spanned >3 multivariate standard deviations according to Leyer and Wesche (2007) using the *vegan* package (Oksanen et al., 2022).

To investigate how functional traits differed between the grasslands and the botanical garden, we performed a principal component analysis (PCA) on scaled and centred data. We checked that the primary axis length was <3 as described in Leyer and Wesche (2007). The significance of each parameter was calculated with the *vegan*‐package (Oksanen et al., 2022). We tested for differences between species means of the three habitats by running linear models (LM) and calculating contrasts on the minimum adequate models using the package *emmeans* (Lenth, 2023). To meet model assumptions, we log‐transformed all dependent variables except for LDMC and leaf C.

To investigate whether there were differences in the timing of the phenological stages between the habitats, we conducted a PCA on the scaled and centred data of the phenological stages. We tested for differences between species means of the three habitats with generalised linear models (GLM) with a Poisson distribution and simplified it to the minimum adequate model by comparing all possible models with the second‐order Akaike Information Criterion from the *MuMIn* package (Bartoń, 2023). If we detected overdispersion, we accounted for it by using a negative binomial distribution with the function *glm.nb* from the *MASS* package (Venables & Ripley, 2002). To further test for statistical differences of the means of phenological stages across habitats, we calculated contrasts of our minimum adequate model using the package *emmeans* (Lenth, 2023).

To investigate whether trait‐phenology relationships are consistent across different habitats or whether they depend on habitat conditions, we used boosted regression trees (BRT) using the R package *gbm* (Greenwell et al., 2019, modifications by Elith et al., 2008). For each of the abovementioned phenological stages, we ran a separate model with the phenological stage as the dependent variable and all morpho‐physiological traits (Table 3) and habitat conditions (aspect, inclination, soil depth, pH, electric conductivity, soil C:N ratio, soil N content, soil moisture, leaf area index, temperature, relative humidity) as explanatory variables.

We investigated the association between traits, habitat conditions and phenological stages in separate models for MG and SDG to show how the within‐habitat associations differ across habitats, i.e., if the same traits are influential predictors of the same phenological stages between the habitats. This approach allowed us to assess the most important drivers of phenology within each of the habitats and compare if they differed across habitats.

Thus, BRTs for each phenological stage were run twice: one model each for MG and SDG. Since we only had one bed per species in BG, we were not able to run a separate model for BG.

Each model used a learning rate of 0.001, a bagging fraction of 0.75, a tree complexity of 2 and an adequate error distribution for the type of data. To fit the models, we used the function *gbm.step* taking the cross‐validation correlation (*cv*) as a measure of fit. To retrieve the most parsimonious model, we used the *gbm.simplify*, which reduces predictor variables that do not lead to a significant decrease in *cv*. Parameters with <2% relative influence were further removed to improve ecological interpretation. Furthermore, we determined the direction of influence by interpreting the partial dependence plots (PDPs) provided from the function *gbm.plot* for each independent parameter of the simplified model (cf. Supporting Information).

## 3 RESULTS

### 3.1 Differences in habitat conditions

**FIGURE A2:**
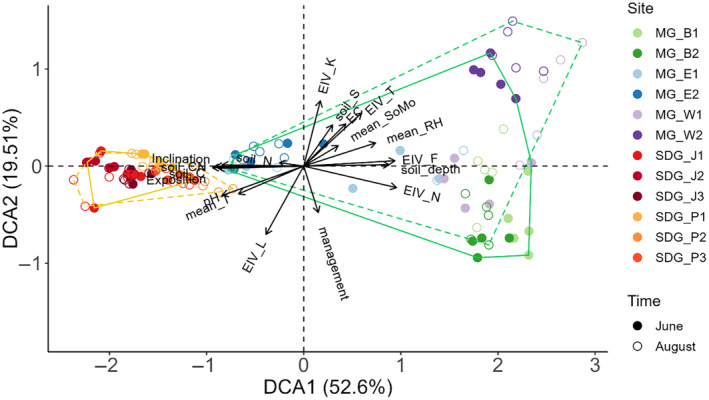
DCA of the vegetation surveys in June and August, respectively. Solid line polygons represent all surveys from June while dashed line polygons display records from August. The arrows represent significant fitted environmental parameters (*p* < .05). EC, electric conductivity of the soil; EIV_F, EIV for soil moisture; EIV_K, EIV for continentality; EIV_L, EIV for light; EIV_N, EIV for nutrients; EIV_T, EIV for temperature; mean_RH, mean relative air humidity; mean_SoMo, mean soil moisture; mean_T, mean air temperature; soil_C, soil carbon content; soil_CN, ratio of soil carbon and nitrogen content; soil_N, soil nitrogen content. Details on the environmental parameters are described in the text.

BG, MG and SDG significantly differed in most habitat conditions: the largest differences were found between SDG compared to BG and MG while BG–MG showed overall more similarities (Table A1, Note A2). Accordingly, the results of the DCA on the vegetation surveys (Figure A2) revealed that MG and SDG were characterised by different species, leading to the differentiation of the two clusters. While the species composition of MG was rather heterogeneous, SDG showed a homogenous species composition across all plots and sites. The groupings in the DCA were linked to inclination, aspect, soil nutrients, mean temperature and the soil pH value, which were higher on SDG, compared to MG. Soil depth and the mean relative humidity, as well as the EIV for moisture and nutrients, were higher on MG and led to an even stronger distinction along DCA1. Mainly associated with DCA2 were management, EC, overall soil moisture, soil sulphur content and the EIV for continentality, light and temperature.

### 3.2 Differences in morpho‐physiological traits between habitats

**FIGURE 1:**
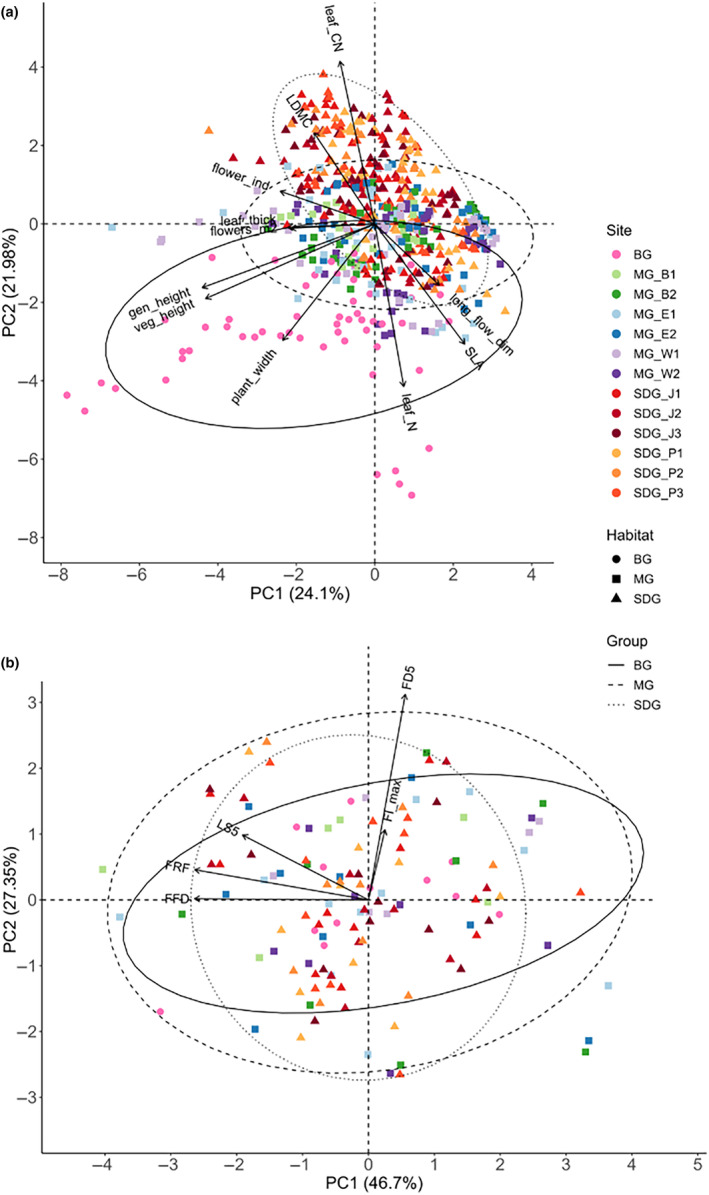
PCAs for all species and sites, showing (a) the measured functional traits including plant‐, leaf‐, and floral traits, and (b) the phenological stages. Note that LS_50_ was excluded because of too few species reaching it. The ellipses show the habitat grouping with 95% confidence while the arrows represent significant fitted parameters (*p* < .05). BG, botanical garden; MG, mesophilic grassland; SDG, semi‐dry grassland. Trait abbbreviations: flower_ind, flowers per individual; flowers_m2, flower density per square meter; gen_height, generative height; LDMC, leaf dry matter content; leaf_C, leaf carbon content; leaf_CN, ratio of leaf carbon and nitrogen content; leaf_N, leaf nitrogen content; leaf_thick, leaf thickness; long_flow_dim, longest flower dimension/flower size; SLA, specific leaf area; veg_height, vegetative height. Phenological stages: FD5, 5% flowering duration; FFD, first flowering day; FI_max_, maximum flower intensity; FRF, day of the first ripe fruit; LS5, day of reaching 5% leaf senescence; LS50, day of reaching 50% leaf senescence.

**FIGURE A3:**
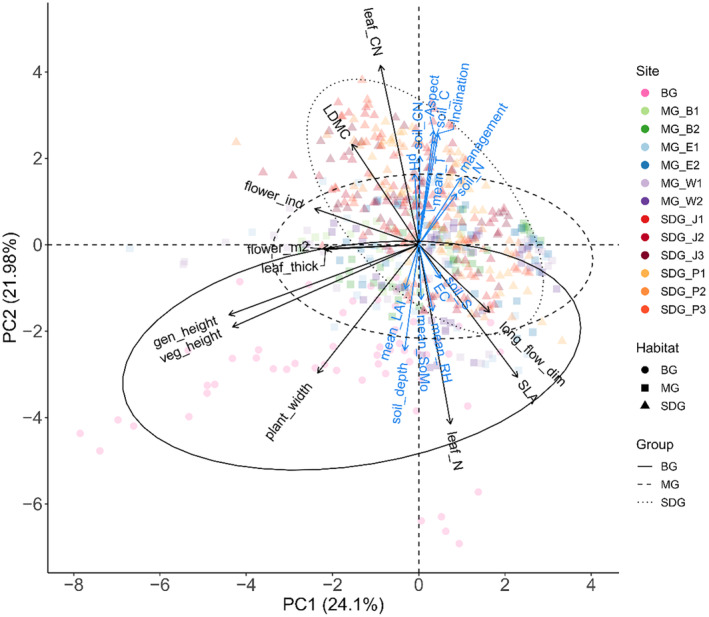
PCA for all species and sites, showing the measured functional traits including plant‐, leaf‐ and floral traits. The ellipses show the habitat grouping with 95% confidence while the arrows represent significant fitted parameters (*p* < .05, black = traits, blue = environmental conditions). BG, botanical garden; MG, mesophilic grassland; SDG, semi‐dry grassland. Trait abbreviations: flower_ind, flowers per individual; flowers_m2, flower density per square meter; gen_height, generative height; LDMC, leaf dry matter content; leaf_C, leaf carbon content; leaf_CN, ratio of leaf carbon and nitrogen content; leaf_N, leaf nitrogen content; leaf_thick, leaf thickness; long_flow_dim, longest flower dimension/flower size; SLA, specific leaf area; veg_height, vegetative height. For details, see Section 2.

**FIGURE A4:**
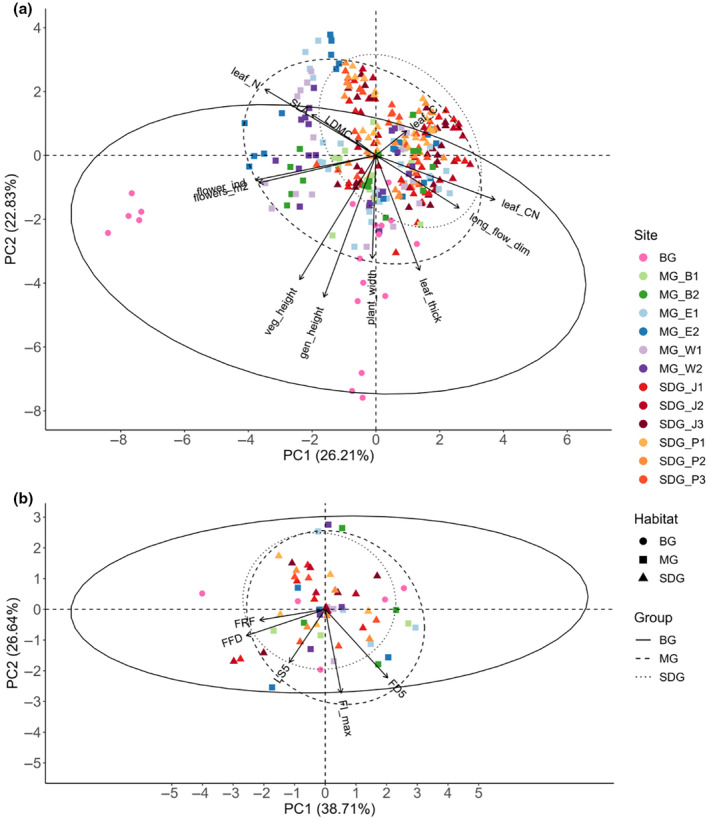
PCAs for the match species and all sites, showing (a) the measured functional traits including plant‐, leaf‐ and floral traits and (b) the phenological stages. Note that LS_50_ was excluded because of too few species reaching it. The ellipses show the habitat grouping with 95% confidence while the arrows represent significant fitted parameters (*p* < .05). BG, botanical garden; MG, mesophilic grassland; SDG, semi‐dry grassland. Trait abbbreviations: flower_ind, flowers per individual; flowers_m2, flower density per square meter; gen_height, generative height; LDMC, leaf dry matter content; leaf_C, leaf carbon content; leaf_CN, ratio of leaf carbon and nitrogen content; leaf_N, leaf nitrogen content; leaf_thick, leaf thickness; long_flow_dim, longest flower dimension/flower size; SLA, specific leaf area; veg_height, vegetative height. Phenological stages: FD5, 5% flowering duration; FFD, first flowering day; FI_max_, maximum flower intensity; FRF, day of the first ripe fruit; LS5, day of reaching 5% leaf senescence; LS50, day of reaching 50% leaf senescence.

All three habitats differed in their morpho‐physiological plant trait compositions (Figure 1a). BG displayed the largest variation in traits along PC1 (24.1% explained variance), while PC2 (21.98% explained variance) mainly captured differences between the three habitats. BG was more similar to MG than to SDG; there was a strong overlap in trait composition between MG and SDG. While PC1 was related to generative and vegetative height, leaf thickness and flower density per square meter, PC2 was related to leaf N, LDMC, SLA, leaf C:N ratio and flower size. The differences in traits between habitats were mainly linked to soil depth and nutrients, inclination and aspect along PC2 (Figure A3). When conducting a PCA with only match species (Figure A4a), the overall pattern remained largely consistent although the importance of traits to explain the variance of PC1 and PC2 partly changed.

**FIGURE 2:**
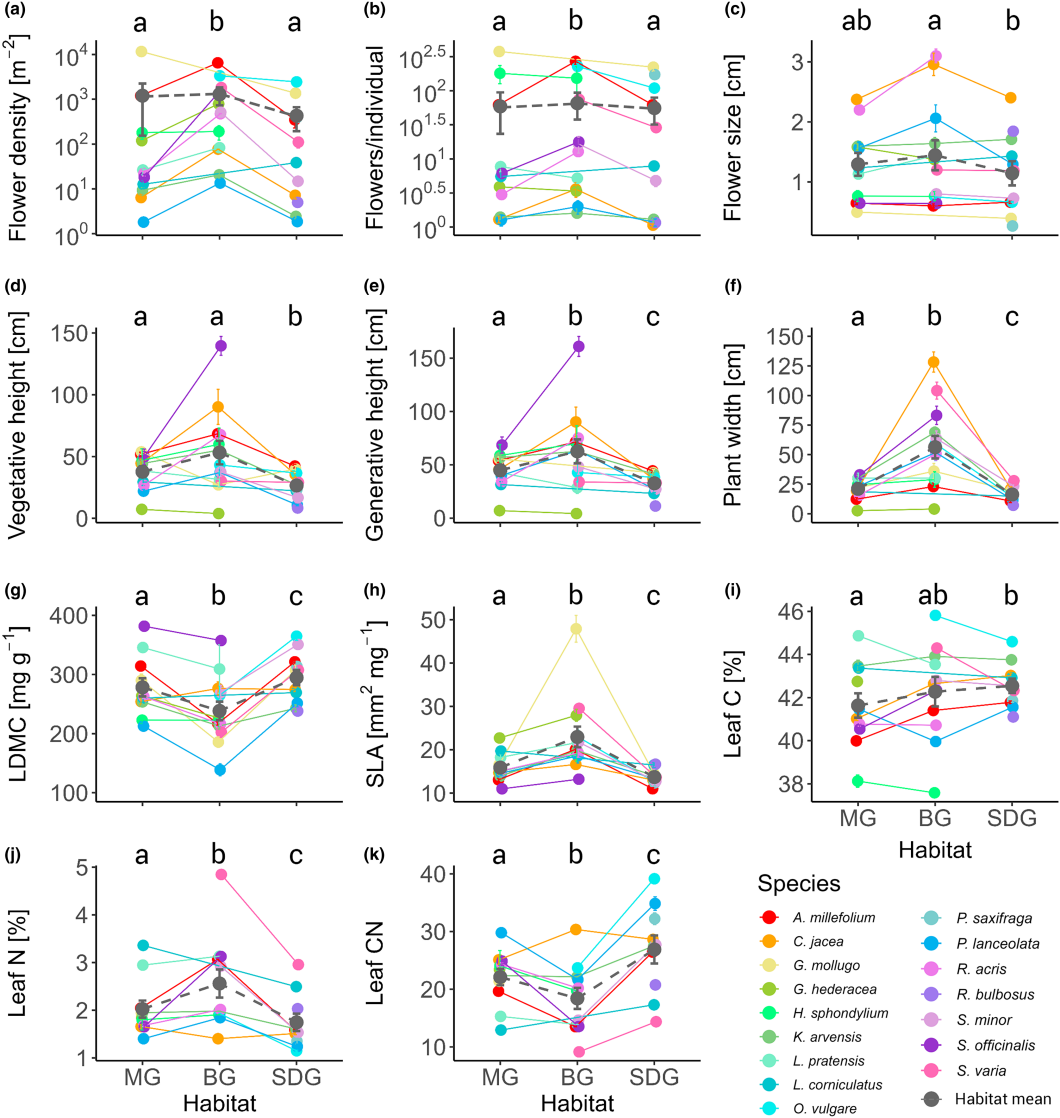
Species‐specific differences in morpho‐physiological traits between the botanic garden (BG), mesophilic (MG) and semi‐dry grassland (SDG). Each point represents a species mean of the respective habitat. The colour code corresponds to the species in the legend while the grey points with dashed lines are the habitat means with standard error. Different letters on top indicate statistically different results of the habitat means based on contrasts as described in the methods.

The contrasts of the LMs showed that trait means differed across most traits and habitats (Figure 2a–k, Table A3). More specifically, compared to MG and SDG, the species in BG tended to be taller (BG‐MG‐SDG: vegetative height: 53.2‐37.8‐26.6 cm, generative height: 62.6‐44.9‐32.6 cm) and broader (plant width: 56.3‐21.1‐16.3 cm) and had higher leaf N (2.6‐2.0‐1.8%) and SLA (23.0‐15.9‐13.7mm^2^/mg) and lower leaf C (42.3‐41.6‐42.6%) and LDMC (238.5‐278.3‐294.8 mg/g). Details on the model outputs and contrasts can be found in Tables A2 and A3.

### 3.3 Differences in flowering and autumn phenology between habitats

**FIGURE 3:**
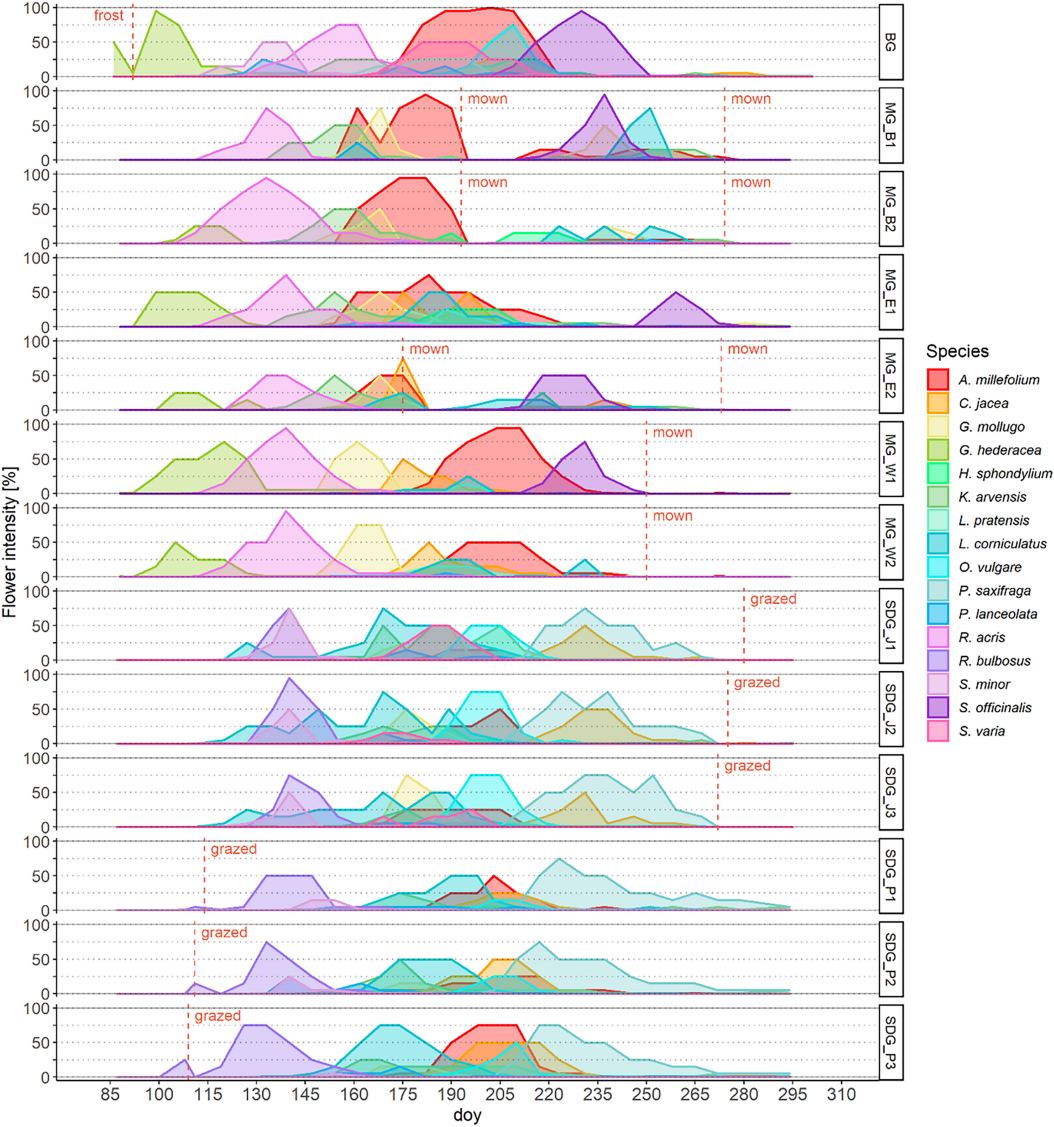
Flower intensity [%] over the period of observation, expressed in days of the year (doy) for each site and species. The vertical dashed lines give information on management events or in case of the botanical garden (BG), displaying early‐year frost events. Different colours represent the species as shown in the legend. For a detailed description of the sites, see Table 2.

The flowering patterns in BG seemed to overlap more strongly with the ones on MG than on SDG (Figure 3). The sequence of flowering species mostly differed between the two grassland habitats: On MG segregation was apparent which was displayed by a subsequent flowering of the species. On SDG, there was no clear order of flowering species but rather a co‐occurring flowering with an overall lower flowering intensity. Furthermore, the same species showed different flowering patterns on different sites. Match species showed a generally earlier peak flowering on MG than on SDG, while BG peaked between both.

The PCA on phenological stages for all species showed no clear differentiation across habitats (Figure 1b; explained variance PC1: 46.7%, PC2: 27.35%). When comparing the phenological stages for the match species only, there was no clear pattern either across habitats (Figure A4b).

**FIGURE 4:**
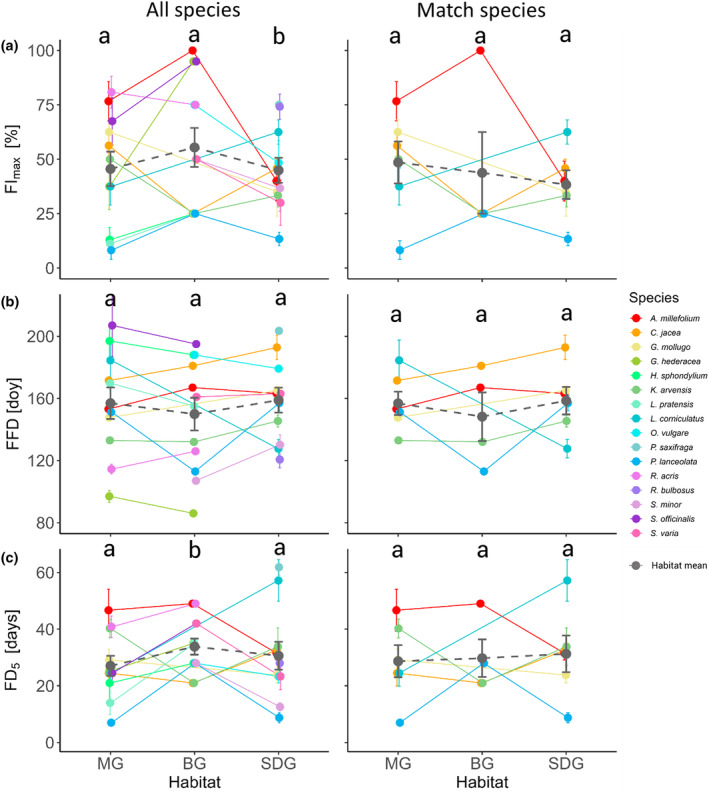
Species‐specific differences in flowering phenology stages between the botanic garden (BG), mesophilic (MG) and semi‐dry grassland (SDG). Shown are comparisons for all species (left column), and six match species (right column) for (a) maximum flower intensity (FI_max_), (b) first flowering day (FFD), and (c) flowering duration with at least 5% open flowers (FD_5_). Each point represents a species mean of the respective habitat. The colour code corresponds to the species in the legend while the grey points with dashed lines are the habitat means with standard error. Single species in BG cannot have a standard error by definition due to one species bed only. Different letters on top indicate statistically different results of the habitat means based on contrasts as described in the methods.

**FIGURE 5:**
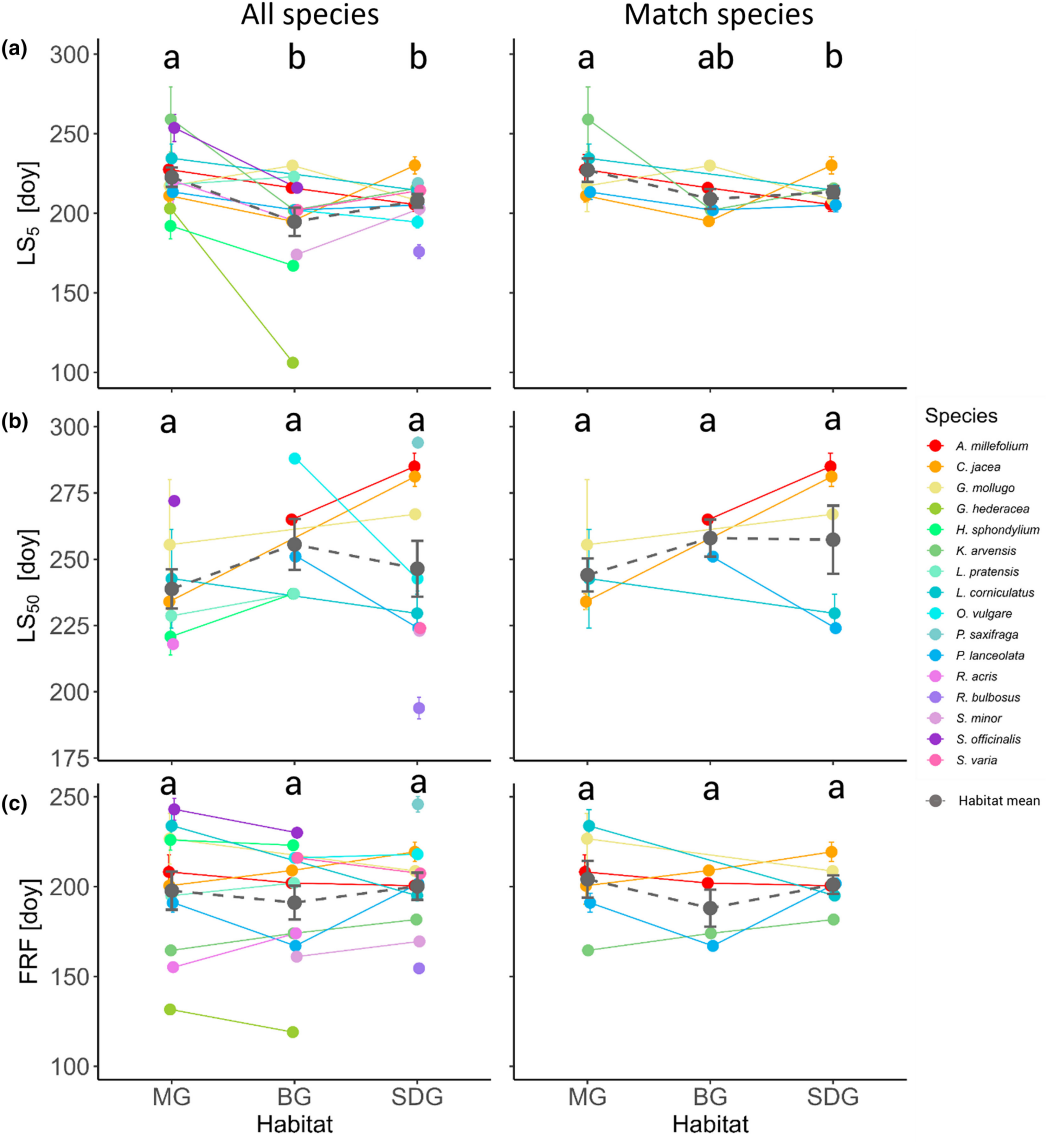
Species‐specific differences in autumn phenology stages between the botanic garden (BG), mesophilic (MG) and semi‐dry grassland (SDG). Shown are comparisons for all species (left column), and six match species (right column) for (a) day of reaching 5% leaf senescence (LS_5_), (b) day of reaching 50% leaf senescence (LS_50_), and (c) day of the first ripe fruit observed (FRF). Each point represents a species mean of the respective habitat. The colour code corresponds to the species in the legend while the grey points with dashed lines are the habitat means with standard error. Single species in BG cannot have a standard error by definition due to one species bed only. Different letters on top indicate statistically different results of the habitat means based on contrasts as described in the methods.

The GLMs showed that there were strong species‐specific differences for both all species and match species between the habitats in the timing of phenological events (Figures 4 and 5, GLM outputs are provided in Tables A4 and A5, details on the contrasts in Table A6). Some species (e.g., *Centaurea jacea* and *Knautia arvensis* ) flowered around 10%–30% more intensively in their natural habitats compared to BG (Figure 4). In contrast, *Glechoma hederacea*, *Sanguisorba officinalis* and *Achillea millefolium* peaked at 25–60% lower FI_max_‐values in their natural habitats than in BG. The GLMs and subsequent contrasts of the flowering phenological stages for all 16 species (Figure 4, Table A6) showed that mean FI_max_ on SDG was slightly lower than in BG (10%, *p* < .05) and that mean FD_5_ in BG was higher than on MG (ca. 6 days, *p* < .01) or SDG (ca. 3 days, *p* < .05). For the match species, habitat did not have a significant influence on flowering phenological stages.

Comparing autumn phenological stages, we only found a later mean LS_5_ on MG, compared to BG (31 days, *p* < .001) and SDG (15 days, *p* < .01) across all species (Figure 5, Tables A4 and A6). For the match species, we found that mean LS_5_ was not species‐specific but on average happened slightly later on MG, compared to SDG (14 days, *p* < .05, Figure 5). We did not detect any statistically significant differences between habitats for match species for the other phenological stages (Table A5).

### 3.4 Trait‐phenology relationships across different habitats

**FIGURE 6:**
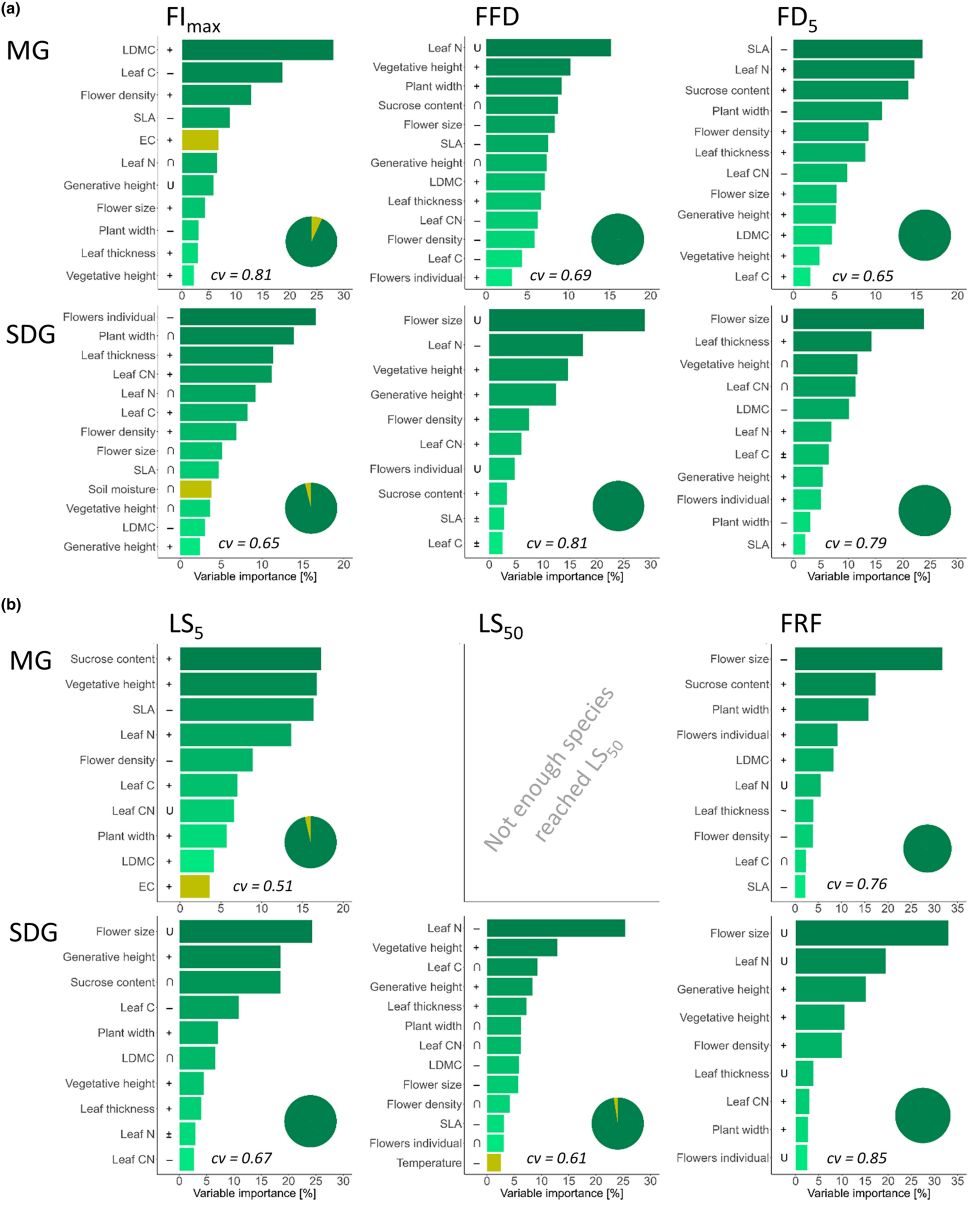
Simplified Boosted Regression Trees (BRT) across all species and habitats showing the relative influence of the most important plant functional (green) and environmental traits (yellow) on (a) flowering phenology and (b) autumn phenology. Columns indicate the phenological traits, which are maximum flower intensity (FI_max_), first flowering day (FFD), and 5% flowering duration (FD_5_), and day of 5% leaf senescence (LS_5_), day of 50% leaf senescence (LS_50_), and day of the first ripe fruit (FRF). Symbols next to the bars represent the direction of influence, which are positive (+), negative (−), ambiguous (±), hump‐shaped (∩), ∪ – shaped (∪), and indifferent (~). Pie charts show the sum of relative influences for trait type and habitat. cv = cross‐validation correlation.

The BRTs for MG and SDG revealed that variation in phenology could mainly be explained by morpho‐physiological traits (Figure 6) while the included environmental variables were largely less influential. Here, it was also apparent that the ranking of predictor importance of the models differed between habitats and phenological stage and sometimes even showed opposite influences as revealed by the partial dependency plots (Figures S1–S6): On MG, FI_max_ was mainly influenced by LDMC (28.1%, positive) and leaf C content (18.6%, negative), while FFD was mostly influenced by leaf N content (15.2%, ∪ – shaped) and FD_5_ mainly by SLA (15.7%, negative), leaf N (14.8%, positive) and sucrose content (14.0%, positive; Figure 6a). In contrast, on SDG, reproductive traits explained most (FI_max_: flower per individuals 16.6%, negative; FFD: flower size 28.9%, ∪ – shaped; FD_5_: flower size 23.8%, ∪ – shaped), directly followed by other plant traits (FI_max_: plant width 13.9%, hump‐shaped; FFD: leaf N 17.4%, negative; FD_5_: leaf thickness 14.3%, positive).

**FIGURE A5:**
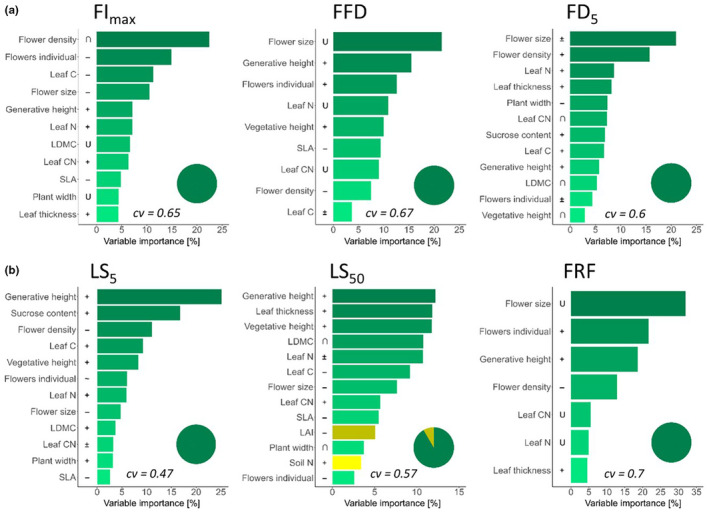
Simplified Boosted Regression Trees (BRT) across all species and all habitats, showing the relative influence of the most important plant functional (green) and environmental traits (yellow) on (a) flowering phenology and (b) autumn phenology. Columns indicate the phenological stages, that are maximum flower intensity (FI_max_), first flowering day (FFD) and 5% flowering duration (FD_5_) and day of 5% leaf senescence (LS_5_), day of 50% leaf senescence (LS_50_) and day of the first ripe fruit (FRF). Symbols next to the bars represent the direction of influence, that are positive (+), negative (−), ambiguous (±), hump‐shaped (∩), ∪ – shaped (∪) and indifferent (~). Pie charts show the sum of relative influences for trait type and habitat. *cv* = cross‐validation correlation.

For autumn phenological stages (Figure 6b), the variation of LS_5_ on MG was mostly explained by sucrose content, vegetative height and SLA (17.3%, 16.7%, 16.4%), while FRF was strongly influenced by flower size (31.7%, ∪ – shaped). Phenological stages on SDG were also mainly explained by reproductive traits, which were flower size (LS_5_: 24.3%, ∪ – shaped; FRF: 33.0%, ∪ – shaped) and generative height (LS_5_: 18.5%, positive; FRF: 15.2%, positive). Only LS_50_ was primarily influenced by vegetative traits, of which leaf N explained most (25.4%, negative). We could not run a separate BRT for LS_50_ on MG because too few species reached LS_50_ in that habitat. BRTs across all habitats showed a similar pattern, and details are provided in Appendix Figure A5 and Note A3.

## 4 DISCUSSION

The study clearly shows that patterns in morpho‐physiological traits differed between botanical gardens, mesophilic grasslands and semi‐dry grasslands while the pattern was less pronounced in phenological stages. The differences in phenology were smaller between the botanical garden and the grasslands than between the grasslands themselves. The results of the BRT analyses showed that across large species sets, plant traits explained the variation in phenological stages, while environmental variables were far less important in a direct comparison. Yet, which traits were most influential strongly changed with the habitat, showing different trait‐phenology relationships acting in different habitats. Phenological responses may be constrained by a suite of certain correlated traits linked to the ecological strategy, which necessarily varies across habitats. Compared to large‐scale studies, we found similar plant ecological strategies with two main dimensions across the plant trait multivariate space, mostly linked to plant height and leaf economics (cf. Díaz et al., 2016). The latter was more associated with the separation of the three habitats, whereas the former tended to drive within‐habitat variation. This indicates that these patterns act similarly on different spatial scales.

### 4.1 Differences in plant traits between habitats

Plant traits related to plant performance, competitive ability and drought resistance (Falster & Westoby, 2003; Garnier, 1992; Gaudet & Keddy, 1988; Pérez‐Harguindeguy et al., 2016), such as leaf N and C:N ratio, plant height, SLA and LDMC strongly varied across habitats. As also shown for the habitat conditions, plant trait composition in BG was more similar to MG than to SDG. This is in accordance with earlier studies on the variation of plant traits linked to environment where the main drivers for differences were light, temperature and nutrient‐ and water stress (Poorter et al., 2009). Higher LDMC values and higher leaf C:N ratio on SDG are both associated with leaf resistance and lifespan and therefore dominate low‐productivity habitats (Garnier, 1992; Pérez‐Harguindeguy et al., 2016), which are expected in a harsh environment such as SDG.

The lower leaf nitrogen concentration of SDG species was likely due to the low‐effective availability of usable nitrogen on SDG on account of the slow mineralisation rate (Ellenberg & Leuschner, 2010). This explanation is underpinned by higher values in SLA, plant height and plant width on MG and BG indicating high productivity and competitive ability (Gaudet & Keddy, 1988; Pérez‐Harguindeguy et al., 2016).

Moreover, differences in plant traits between habitats likely also occurred due to habitat‐specific species which are adapted to the respective habitat conditions. However, we controlled for this by comparing match species only and showed that surprisingly, instead of leaf N, mainly leaf C content, number of flowers and plant size drove the habitat‐specific differences indicating that competitive ability and investment in leaf longevity were the most important processes leading to the observed differences (Falster & Westoby, 2003; Gaudet & Keddy, 1988; Pérez‐Harguindeguy et al., 2016). Since BG and MG were more similar, probably due to irrigation and fertilisation practices in the botanical garden, this similarity of habitat conditions was also reflected in the plant traits of the species where we found intraspecific differences even when focussing on match species only.

The observed differences in trait expression between the botanical garden and grasslands could also be explained by phenotypic plasticity or genetic differences between populations. However, Karbstein et al. (2020) showed that within‐habitat heterogeneity explained intraspecific trait variation much better than genetic diversity, suggesting that also in our dataset, habitat conditions might have been the main drivers of trait variation. In further studies, one could collect seeds from the natural populations and grow them in the botanical garden to account for the genetic component of intraspecific variation.

Our results show that plant strategies regarding morpho‐physiological traits on a local scale are similar to the ones on a global scale (cf. Díaz et al., 2016). Similar to the global pattern, in our study, plant height and leaf economic spectrum (e.g., leaf N, SLA, LDMC) represented two separate dimensions of plant ecological strategies. Across habitats, mainly leaf economics seemed to lead to distinctions while within habitats, plant height mostly drove the separation. This indicates that environmental filtering for those traits favours specific plant ecological strategies, acting similarly on different scales.

### 4.2 Differences in flowering and autumn phenology between habitats

Habitats were less clearly differentiated in their phenology patterns compared to the other functional traits, despite the differences in habitat conditions and vegetation composition. Yet, flowering intensity over the year was more similar between BG and MG, compared to SDG. BG and MG largely showed temporal segregation of species regarding their peak flowering. Füllekrug (1969) also observed higher flowering intensities and shorter flowering times in mesophilic grasslands. On MG, species strongly compete for light, thus these patterns may indicate a temporal avoidance of competition leading to a consecutive flowering pattern. In turn, it reduces competition for pollinators and hence reproductive handicaps (Morales & Traveset, 2008; Mosquin, 1971). The SDG sites generally displayed more synchronous and less intense flowering patterns, which is consistent with previous studies observing a flowering synchronisation without a predominating species at one time but rather evenly distributed flowering intensities (Fantinato et al., 2016; Füllekrug, 1969). Compared to MG, less competition for light on SDG promotes a spatiotemporal co‐occurrence of many species and thus favours flowering synchrony.

Generally, the observation of the durations and intensities of phenological stages was depending on the number and timing of management. Ideally, all sites would need to be managed at similar times and with similar intensities, which was, unfortunately not possible in our observational experiment. For this, controlled experiments such as the Global Change Ecosystem Facility (GCEF; Schädler et al., 2019) provide a suitable basis but again come along with other difficulties such as less established and partly artificial plant communities. Alternatively, not‐investigated factors such as small‐scale variations in climate or soil might be needed and considered in further studies to finally predict the intraspecific differences in phenological stages between species.

We found mostly species‐specific differences in the timing of phenological events between habitats, while differences in the mean values were rare. Still, we found slightly higher FI_max_ and longer FD_5_ in BG and later LS_5_ on MG were visible. This is in line with previous findings showing that responses in phenology can be both, highly trait and species‐specific (Bucher & Römermann, 2020, 2021; Peñuelas et al., 2004). However, phenological responses might also be restricted by certain correlated traits which come along a specific ecological strategy. Since plant strategies necessarily differ across habitats due to the different environmental conditions which could lead to similar phenological responses across habitats.

The species‐specific responses we found for all species were also mirrored when focusing on the match species only with single species having a major influence on the overall pattern (e.g., *L. corniculatus* or *Plantago lanceolata*). Despite the relatively small number of match species, our results hint to habitat‐specific differences which, however, should be further investigated with a higher number of species. These species‐specific phenological responses make community‐level responses less predictable. Since botanical gardens usually study (many) individual species instead of natural plant communities it is important to take habitat conditions into account when studying phenology in botanical gardens, especially when focussing on a small set of species only. Overall, these results suggest that habitat conditions are important when focussing on selected species only. When analysing phenology data on the level of communities though, habitat conditions seem to be less important. Thus, in these approaches, botanical garden studies on many species seem to produce reliable and transferable results. Further studies are needed to also widen the habitats included, as here we only focussed on open habitats. The reliability of forest species might be reduced as typical forest conditions might be less represented in botanical gardens compared to open grassland conditions.

### 4.3 Trait‐phenology relationships across different habitats

Our findings show that the most influential morpho‐physiological traits for phenological stages differed depending on the habitat. That way, phenological responses may be constrained by a set of correlated traits due to different ecological plant strategies, which necessarily vary across habitats. Furthermore, we found that morpho‐physiological functional traits are robust predictors for phenology and therewith confirm the findings from a local scale study by Liu et al. (2021), from a global scale study by König et al. (2018), a botanical garden study by Sporbert et al. (2022) and along elevational gradients by Bucher et al. (2018) and Bucher and Römermann (2021). Consistent with Bucher et al. (2018) and Sporbert et al. (2022), our BRT analyses including all habitat conditions and plant traits indicated that the latter explained phenological responses generally better than included habitat conditions. This raises a classic chicken and egg problem because plant traits are directly affected by and therefore basically mediate the environment. Yet, in a direct comparison of plant traits and habitat conditions in the BRT models, plant traits explained more of the variance which might suggest that they capture environmental variation plus morpho‐physiological characteristics of the plants which together are better predictors for phenological responses than environment alone.

The plant traits being most influential for phenological stages depended on both phenological stage and habitat. In the BRTs including all habitats, reproductive traits, such as flower density or flower size, were very important predictors for all phenological stages. When looking specifically into patterns of SDG, reproductive traits also influenced flowering and autumn phenology most, usually followed by vegetative traits (e.g., vegetative height, leaf nutrients, leaf thickness). This pattern indicates that in habitats where flowering synchrony is more pronounced and hence competition for pollinators, reproductive traits seem to be especially important in mediating phenological responses. In contrast, in habitats where competition for light and therefore temporal segregation of flowering phenology is more pronounced, such as on MG, vegetative traits were the most influential mediators of phenological responses.

Plants flowered earlier with increasing values of productivity‐related traits, such as SLA and leaf N and later with increasing values of resistance‐linked traits, such as LDMC and leaf thickness, which is consistent with previous findings (Bucher et al., 2018; Liu et al., 2021; Sporbert et al., 2022; Sun & Frelich, 2011). Furthermore, we confirmed that an increase in competitive characteristics, such as vegetative and generative height and plant width, led to later flowering (Bolmgren & Cowan, 2008; Liu et al., 2021; Sun & Frelich, 2011).

A similar pattern could be observed for autumn phenology (LS_5_, LS_50_, FRF): most influential plant traits depended on both the phenological stage and habitat. Our findings confirm previous results showing that plant traits are also robust mediators of environmental change for autumn phenological stages (Bucher & Römermann, 2021). Reproductive traits were the most important predictors for autumn phenological stages which indicates that the end of the reproductive phase determines the senescence of plants, which was most visible on SDG. Specifically, the positive relationship between generative height and day of leaf senescence across all habitats, i.e., senescence occurs later with larger generative heights, suggests plant strategies that invest in dispersal and reproductive potential and have and extended carbon acquisition over a growing season. In contrast, the negative relation of leaf N and LS_50_ on SDG suggests a trade‐off between leaf economics and therefore photosynthetic potential and carbon acquisition by having a shorter growing period in drier and harsher environments. Further research is needed to identify possible reasons for this pattern.

### 4.4 Data transferability from botanical gardens to more natural habitats

Our findings that SDG and BG were more different than MG and BG suggest that the phenological data recorded in a botanical garden only partly represents the phenology in the species' natural habitat and rather depends on the environment. The species‐specificity in our study clearly shows that only multi‐species analyses seem to be transferable from botanical gardens to the natural habitat. Single species show contrasting behaviour, which is why studies with only a few species in a botanical garden should always consider the natural habitat or habitat conditions as covariates. When performing analyses involving a broad set of species, however, habitat may be neglected due to the species‐specific balancing effect.

However, plant traits did vary between the investigated habitats and also the BRT analyses showed that traits influenced phenology in different strength and direction depending on the habitat. Therefore, the natural habitat or habitat conditions should not be neglected when investigating plant traits or when linking those to phenology.

### 4.5 Concluding remarks

Data on habitat‐specific differences for plant traits or phenology are rare. We, therefore, suggest focussing on specific parameters of interest and increasing the number of species to overcome species‐specific balancing effects and identify more general patterns in further studies.

Especially autumn phenology has been largely neglected so far but has shown high potential of being a good predictor for changing environments since we found habitat‐specific correlations with plant traits, suggesting that senescence and fruiting might be affecting the phenological patterns in these different habitats.

In particular, reproductive traits seemed to be important predictors of both flower and autumn phenology for all habitats. Only on MG, plant and leaf traits were more influential predictors, but reproductive traits still complemented them. Therefore, we suggest that further studies on phenology should include reproductive traits as they seem to be important mediators of the environment.

Based on our findings, we encourage to conduct even more studies using plant traits as predictors of phenology to bridge the gap and deepen our ecological understanding of plants' interactions with their environment. Including different habitats, such as the understorey in forests, will furthermore reveal the extent to which the detected patterns are generally applicable and when habitat conditions alter the phenological response of plant species. Our results so far suggest that phenological data recorded in a BG are, on average, similar to the phenology of species in their natural environment and can thus provide a solid and efficient basis for large‐scale phenological experiments.

## AUTHOR CONTRIBUTIONS

**Till J. Deilmann:** Formal analysis (lead); investigation (lead); methodology (equal); software (lead); visualization (equal); writing – original draft (lead); writing – review and editing (equal). **Josephine Ulrich:** Investigation (supporting); methodology (equal); supervision (supporting); writing – review and editing (supporting). **Christine Römermann:** Conceptualization (lead); funding acquisition (lead); methodology (equal); resources (lead); supervision (lead); writing – review and editing (equal).

## CONFLICT OF INTEREST STATEMENT

The authors declare no competing interests.

## Supporting information


Data S1.

## Data Availability

The data and code used in this study are available in the data repository of the German Centre for Integrative Biodiversity Research (iDiv) at http://idata.idiv.de/ddm/Data/ShowData/3523.

## Note A1: Coping with missing data of ONSET Hobo v2 Pro logger

We coped with missing values by predicting these from the other available data, as follows. On MG, two sites, and therefore, two data loggers were always located quite close to each other resulting in similar temperature and humidity data. On SDG, this was the case with each three data loggers. We used the best‐fitting linear regression model to predict these data based on the other data logger as a reference. If data was missing on all of the nearby data loggers, due to, e.g., management events, an overall mean within the habitat was calculated as reference data to predict the deviance of each single site in a linear model. On SDG, we could follow a slightly different and more accurate approach because data loggers at the ground had been installed previously. The deviance between these data and the existing data of the 1.5 m data loggers was taken as a reference to predict missing values to that point of time when the ground loggers were installed. Only in the case of data gaps which also occurred in the ground loggers, e.g., for management reasons, the habitat mean was calculated as reference and the deviance to the individual sites was predicted in a linear model. All model assumptions were met and the correlation between the taken data sets was usually very high (*R* ^2^ > .9).

## Note A2: Details on environmental conditions

For almost all environmental conditions, SDG was significantly different from the other two habitats, and for many factors, all three habitats were different. A detailed overview is presented in Table A1.

Regarding the fixed environmental conditions, aspect was not present on MG and in BG and inclination was 0° because these sites were even. In contrast, all SDG sites were south or south‐west exposed slopes with a moderate mean inclination of 15.44 degrees. Within SDG, the sites showed considerable variation regarding both parameters at a first glance. However, despite some significant differences in the aspect between some sites (*F*_5,24_ = 40.02, *p* < .001), the overall cardinal direction was the same, namely south to south‐west. The slope also differed between some sites (*F*_5,24_ = 14.43, *p* < .001), with the Pennickental sites being slightly steeper than those at the Jenzig. Soil depth differed greatly and significantly between habitats (*χ* ^2^ = 63.19, df = 2, *p* < .001), with MG having the deepest soils with >100 cm, followed by BG, which showed the greatest variation. SDG had the shallowest soils with on average less than 10 cm depth.

The slightly alkaline pH values were highest on SDG, followed by BG and MG (*χ* ^2^ = 52.09, df = 2, *p* < .001). The electric conductivity (EC) was highest in MG, while the soils of BG and SDG showed lower values and were only marginally significantly different from each other (*χ* ^2^ = 49.01, df = 2, *p* < .001; BG‐SDG: *p* = .083). Regarding the soil nutrient content, MG had the lowest C:N ratio, followed by BG and then SDG (*χ* ^2^ = 56.94, df = 2, *p* < .001). BG soil contained the least and SDG soil the most nitrogen (*F*_2,70_ = 10.92, *p* < .001), but the latter also had highest carbon contents (*χ* ^2^ = 52.62, df = 2, *p* < .001), resulting in the highest C:N ratio (*χ* ^2^ = 56.95, df = 2, *p* < .001). The average soil moisture varied significantly between all three habitats, with MG having the highest overall soil moisture (*χ* ^2^ = 62.77, df = 2, *p* < .001). BG and SDG were more similar to each other, but still SDG had the driest soils on average over the year. Regarding the leaf area index (LAI), SDG had the lowest overall mean value, and differed significantly between BG and MG (*χ* ^2^ = 20.48, df = 2, *p* < .001). Air temperature and relative air humidity varied strongly across habitats throughout the year. Not surprisingly, the driest time was during summer (day of the year ca. 175–265, i.e., 24 June to 22 September) where daily mean temperatures normally exceeded daily mean relative humidity.

While temperature showed an overall unimodal trend, relative humidity did not show a real trend but rather monthly fluctuations. For statistical comparisons between habitats, we only used the data which was present for all three habitats to avoid distorted values. Regarding the overall mean temperature only, no significant differences between habitats could be found (*χ* ^2^ = 2.12, df = 2, *p* = .346). Nevertheless, the warmest habitat on average was BG with 15.84°C, followed by SDG and MG with slightly lower temperatures. Minimum daily mean temperature was highest in BG with 5.72°C while MG and SDG were up to one degree colder. In contrast, maximum daily mean temperature was highest on SDG with 27.66°C which was more than one degree warmer than in BG and MG.

On the other hand, relative humidity was on average highest on MG being significantly different from the two other habitats (*F*_2,2635_ = 40.05, *p* < .001), and lowest on SDG differing marginally significantly from BG (*p* = .064). Additionally, SDG had the lowest daily mean minimum humidity by far as well with just half of the minimum values of the other two habitats. At the same time, the maximum daily humidity was highest on SDG almost reaching 100%.

The abundance weighted Ellenberg indicator values (EIV) mainly mirrored the abovementioned habitat differences in environment conditions. MG and SDG differed significantly in all calculated mean EIV apart from the soil reaction. The SDG sites had mostly smaller values than MG, including the temperature value (Wilcoxon‐Rank‐Sum‐test: *W* = 795, *p* < .001), and only the light value exceeded that of MG (*W* = 184, *p* < .001). Based on EIV only, SDG was a nitrogen poor, relatively dry, moderately warm, and light habitat. On the other hand, MG was characterised as an intermediate nutrient rich, medium‐moist, moderately warm, and light environment.

The PCA across all sites regarding environmental variables showed three distinct groups separating the three habitats. The position of the sites along PC 1 indicated that MG and BG were more similar to each other than to SDG. PC1 was positively related with mean temperature, soil pH, soil C:N ratio, aspect, inclination, and soil carbon content, and negatively linked to soil depth, mean relative air humidity, and mean soil moisture.

Within the habitats, there was also variation between the single sites, mainly displayed along PC2. The sites mainly differed in LAI, soil nitrogen and sulphur content, and electric conductivity. The Pennickental (SDG_P) and Jenzig (SDG_J) sites differed mainly in mean temperature, relative humidity, soil N and pH while the distinction of sites within one location was explained by mean soil moisture, LAI, soil C, and inclination.

## Note A3: Description of Boosted Regression Tree analysis across all habitats

Results of the first BRT models across all habitats (BG, MG, SDG) revealed that variations in phenology could mostly be explained by plant traits. The impact of habitat conditions was not substantial except for the model on LS_50_ in which LAI and soil nitrogen explained together 8.4% of the variation of LS_50_ (Figure A5b). Overall, flower size, flower density, flowers per individual, and generative height were consistently important to explain most phenological stages of both flowering and autumn phenology. The most important drivers of FI_max_ were flower density (22.5%, hump‐shaped relationship) and flowers per individual (14.9%, negative), while the most influential traits for the other phenological stages were flower size (21.5%, ∪ – shaped) and generative height (15.5%, positive) for FFD, and flower size (21.0%, ambiguous) and flower density (15.7%, positive) for FD_5_ (Figure A5a). An important predictor for autumn phenological stages across all habitats (Figure A5b) was generative height with a consistently positive relationship (LS_5_: 25.2%; LS_50_: 12.2%, FRF: 18.6%). Other reproductive traits also explained a considerable amount that was sucrose content (LS5: 16.8%, positive), flower size (FRF: 32.0%, ∪ – shaped) and flowers per individual (FRF: 21.7%, positive).

**TABLE A1:** Descriptive table of the measured environmental conditions for all three observed habitats.

Parameter	Test‐values	BG	MG	SDG
Aspect [°]	*χ* ^2^ = 65.74, df = 2, *p* < .001	0	0	178.93 ± 1.77
		**a**	**a**	**b**
Inclination [°]	*χ* ^2^ = 65.73, df = 2, *p* < .001	0	0	15.44 ± 0.48
		**a**	**a**	**b**
Soil depth [cm]	*χ* ^2^ = 63.19, df = 2, *p* < .001	84.8 ± 8.63	100	8.96 ± 0.42
		**a**	**b**	**c**
pH	*χ* ^2^ = 52.09, df = 2, *p* < .001	7.7 ± 0.04	7.48 ± 0.01	7.87 ± 0.01
		**a**	**b**	**c**
EC [μS/cm]	*χ* ^2^ = 49.01, df = 2, *p* < .001	206.62 ± 11.73	339.33 ± 14.66	224.07 ± 3.34
		**a**	**b**	**a**
soil C:N ratio	*χ* ^2^ = 56.95, df = 2, *p* < .001	18.33 ± 1.18	12.93 ± 0.42	29.51 ± 0.78
		**a**	**b**	**c**
soil N content [%]	*F*_2,70_ = 10.92, *p* < .001	0.27 ± 0.02	0.34 ± 0.01	0.35 ± 0.01
		**a**	**b**	**b**
Soil moisture mean [% vol]	*χ* ^2^ = 62.77, df = 2, *p* < .001	17.86 ± 0.82	21.84 ± 0.52	17.02 ± 0.55
		**a**	**b**	**c**
Soil moisture min [% vol]		2	7.16	6.14
Soil moisture max [% vol]		34.72	41.42	40.64
LAI mean	*χ* ^2^ = 20.48, df = 2, *p* < .001	1.92 ± 0.16	1.91 ± 0.09	1.3 ± 0.06
		**a**	**a**	**b**
LAI min		0	0.01	0
LAI max		6.73	5.19	4.09
Daily temperature mean [°C]	*χ* ^2^ = 2.12, df = 2, *p* = .346	15.84 ± 0.32	15.33 ± 0.13	15.49 ± 0.13
		a	a	a
Daily temperature min [°C]		5.72	4.94	4.62
Daily temperature max [°C]		26.48	25.94	27.66
Daily rel. humidity mean [%]	*F*_2,2635_ = 44.05, *p* < .001	70.42 ± 0.7	73.13 ± 0.26	68.83 ± 0.38
		**a**	**b**	**a**
Daily rel. humidity min [%]		50.95	49.23	29.45
Daily rel. humidity max [%]		93.84	95.51	99.98
Weighted Ellenberg *N*‐value	*W* = 928, *p* < .001	–	5.51 ± 0.2	2.89 ± 0.05
		–	**a**	**b**
Weighted Ellenberg *L*‐value	*W* = 184, *p* < .001	–	7.02 ± 0.09	7.5 ± 0.03
			**a**	**b**
Weighted Ellenberg *T*‐value	*W* = 795, *p* < .001	–	5.54 ± 0.04	5.21 ± 0.01
			**a**	**b**
Weighted Ellenberg *F*‐value	*W* = 863, *p* < .001	–	4.61 ± 0.12	3.34 ± 0.02
			**a**	**b**
Weighted Ellenberg *R*‐value	*W* = 532, *p* = .339	–	7.32 ± 0.07	7.12 ± 0.12
			a	a
Weighted Ellenberg *K*‐value	*W* = 660, *p* < .01	–	3.29 ± 0.12	2.91 ± 0.05
			**a**	**b**

*Note*: Means are given with the respective standard errors. Test statistics give results of the ANOVA, Kruskal–Wallis Test, and Wilcoxon‐Rank‐Sum test, respectively. Different letters indicate significant differences between habitats as extracted from the pairwise *t*‐test and pairwise Wilcoxon‐Rank‐Sum test, respectively, and are marked in bold. Daily temperature and relative humidity means were calculated by averaging all 24 measurements of a day taken by the Onset HOBO data loggers on each site and grouped per habitat.

Abbreviations: BG, botanical garden; C:N, carbon to nitrogen; EC, electric conductivity; LAI, leaf area index; MG, mesophilic grassland; N, nitrogen; SDG, semi‐dry grassland.

**TABLE A2:** Outputs of the linear models (LMs) across all observed species and habitats.

Plant functional trait	Estimate	SE	*t* Value	*p* Value
*log(Flower density)*				
(Intercept)	**8.54**	**0.41**	**20.81**	**<.001**
HabitatMG	**−2.20**	**0.34**	**−6.54**	**<.001**
HabitatSDG	**−2.73**	**0.34**	**−7.96**	**<.001**
speciesCen_jac	**−4.29**	**0.42**	**−10.28**	**<.001**
speciesGal_mol	**1.92**	**0.41**	**4.68**	**<.001**
speciesGle_hed	**−2.19**	**0.51**	**−4.28**	**<.001**
speciesHer_sph	**−1.85**	**0.66**	**−2.82**	**.006**
speciesKna_arv	**−4.73**	**0.42**	**−11.32**	**<.001**
speciesLat_pra	**−3.47**	**0.66**	**−5.28**	**<.001**
speciesLot_cor	**−2.99**	**0.43**	**−6.96**	**<.001**
speciesOri_vul	**1.47**	**0.48**	**3.03**	**.003**
speciesPim_sax	0.00	0.51	0.01	.994
speciesPla_lan	**−5.69**	**0.41**	**−13.95**	**<.001**
speciesRan_acr	**−3.50**	**0.49**	**−7.15**	**<.001**
speciesRan_bul	**−4.35**	**0.54**	**−7.99**	**<.001**
speciesSan_min	**−3.39**	**0.51**	**−6.67**	**<.001**
speciesSan_off	**−3.16**	**0.54**	**−5.81**	**<.001**
speciesSec_var	**−1.17**	**0.59**	**−2.00**	**.048**
*log(Flowers per individual)*				
(Intercept)	**4.91**	**0.17**	**28.57**	**<.001**
HabitatMG	**−0.70**	**0.14**	**−4.99**	**<.001**
HabitatSDG	**−0.82**	**0.14**	**−5.71**	**<.001**
speciesCen_jac	**−3.97**	**0.17**	**−22.69**	**<.001**
speciesGal_mol	**1.41**	**0.17**	**8.22**	**<.001**
speciesGle_hed	**−3.00**	**0.21**	**−13.97**	**<.001**
speciesHer_sph	**0.71**	**0.28**	**2.58**	**.011**
speciesKna_arv	**−3.92**	**0.17**	**−22.42**	**<.001**
speciesLat_pra	**−2.54**	**0.28**	**−9.25**	**<.001**
speciesLot_cor	**−2.25**	**0.18**	**−12.55**	**<.001**
speciesOri_vul	**0.53**	**0.20**	**2.59**	**.011**
speciesPim_sax	**1.04**	**0.21**	**4.84**	**<.001**
speciesPla_lan	**−4.10**	**0.17**	**−23.98**	**<.001**
speciesRan_acr	**−3.02**	**0.20**	**−14.75**	**<.001**
speciesRan_bul	**−3.95**	**0.23**	**−17.34**	**<.001**
speciesSan_min	**−2.59**	**0.21**	**−12.15**	**<.001**
speciesSan_off	**−2.37**	**0.23**	**−10.42**	**<.001**
speciesSec_var	**−0.72**	**0.24**	**−2.93**	**.004**
*log(Plant width)*				
(Intercept)	**3.37**	**0.12**	**27.29**	**<.001**
HabitatMG	**−0.83**	**0.10**	**−8.31**	**<.001**
HabitatSDG	**−1.13**	**0.10**	**−11.14**	**<.001**
speciesCen_jac	**0.72**	**0.13**	**5.58**	**<.001**
speciesGal_mol	**0.56**	**0.12**	**4.59**	**<.001**
speciesGle_hed	**−1.66**	**0.16**	**−10.52**	**<.001**
speciesHer_sph	**0.45**	**0.20**	**2.24**	**.027**
speciesKna_arv	**0.67**	**0.13**	**5.24**	**<.001**
speciesLat_pra	**0.59**	**0.20**	**2.94**	**.004**
speciesLot_cor	**0.38**	**0.13**	**2.91**	**.004**
speciesOri_vul	**0.44**	**0.15**	**2.97**	**.004**
speciesPim_sax	0.22	0.16	1.40	.166
speciesPla_lan	**0.41**	**0.13**	**3.24**	**.002**
speciesRan_acr	0.26	0.15	1.73	.086
speciesRan_bul	−0.28	0.17	−1.67	.099
speciesSan_min	**0.86**	**0.16**	**5.53**	**<.001**
speciesSan_off	**0.95**	**0.17**	**5.67**	**<.001**
speciesSec_var	**1.13**	**0.18**	**6.29**	**<.001**
*log(Vegetative height)*				
(Intercept)	**4.24**	**0.12**	**35.14**	**<.001**
HabitatMG	**−0.22**	**0.10**	**−2.26**	**.026**
HabitatSDG	**−0.57**	**0.10**	**−5.72**	**<.001**
speciesCen_jac	−0.14	0.13	−1.11	.27
speciesGal_mol	−0.11	0.12	−0.91	.367
speciesGle_hed	**−2.18**	**0.15**	**−14.12**	**<.001**
speciesHer_sph	−0.15	0.20	−0.75	.456
speciesKna_arv	**−0.32**	**0.13**	**−2.53**	**.013**
speciesLat_pra	**−0.51**	**0.20**	**−2.61**	**.011**
speciesLot_cor	**−0.63**	**0.13**	**−4.87**	**<.001**
speciesOri_vul	−0.14	0.15	−0.95	.343
speciesPim_sax	**−0.53**	**0.15**	**−3.43**	**<.001**
speciesPla_lan	**−1.08**	**0.12**	**−8.81**	**<.001**
speciesRan_acr	**−0.64**	**0.15**	**−4.34**	**<.001**
speciesRan_bul	**−1.57**	**0.16**	**−9.60**	**<.001**
speciesSan_min	**−0.86**	**0.15**	**−5.65**	**<.001**
speciesSan_off	−0.05	0.16	−0.29	.77
speciesSec_var	**−0.44**	**0.18**	**−2.50**	**.014**
*log(Generative height)*				
(Intercept)	**4.25**	**0.10**	**42.49**	**<.001**
HabitatMG	**−0.23**	**0.08**	**−2.82**	**.006**
HabitatSDG	**−0.50**	**0.08**	**−5.98**	**<.001**
speciesCen_jac	−0.15	0.10	−1.51	.134
speciesGal_mol	−0.04	0.10	−0.37	.714
speciesGle_hed	**−2.20**	**0.13**	**−17.57**	**<.001**
speciesHer_sph	0.05	0.16	0.29	.769
speciesKna_arv	−0.03	0.10	−0.27	.786
speciesLat_pra	**−0.49**	**0.16**	**−3.04**	**.003**
speciesLot_cor	**−0.62**	**0.10**	**−5.92**	**<.001**
speciesOri_vul	−0.17	0.12	−1.47	.145
speciesPim_sax	−0.23	0.13	−1.87	.064
speciesPla_lan	**−0.37**	**0.10**	**−3.74**	**<.001**
speciesRan_acr	**−0.42**	**0.12**	**−3.48**	**<.001**
speciesRan_bul	**−1.35**	**0.13**	**−10.16**	**<.001**
speciesSan_min	**−0.46**	**0.12**	**−3.70**	**<.001**
speciesSan_off	0.23	0.13	1.71	.089
speciesSec_var	**−0.38**	**0.14**	**−2.66**	**.009**
*log(Flower size)*				
(Intercept)	**−0.34**	**0.05**	**−7.30**	**<.001**
HabitatMG	**−0.09**	**0.04**	**−2.22**	**.029**
HabitatSDG	**−0.12**	**0.04**	**−3.06**	**.003**
speciesCen_jac	**1.33**	**0.05**	**27.75**	**<.001**
speciesGal_mol	**−0.38**	**0.05**	**−7.99**	**<.001**
speciesGle_hed	**0.84**	**0.06**	**14.25**	**<.001**
speciesHer_sph	0.12	0.08	1.64	.105
speciesKna_arv	**0.95**	**0.05**	**19.76**	**<.001**
speciesLat_pra	**0.60**	**0.08**	**8.01**	**<.001**
speciesLot_cor	**0.75**	**0.05**	**15.17**	**<.001**
speciesOri_vul	0.05	0.06	0.96	.341
speciesPim_sax	**−0.87**	**0.06**	**−14.73**	**<.001**
speciesPla_lan	**0.81**	**0.05**	**17.35**	**<.001**
speciesRan_acr	**1.25**	**0.06**	**22.16**	**<.001**
speciesRan_bul	**1.07**	**0.06**	**17.19**	**<.001**
speciesSan_min	**0.14**	**0.06**	**2.41**	**.018**
speciesSan_off	−0.02	0.06	−0.31	.761
speciesSec_var	**0.61**	**0.07**	**9.06**	**<.001**
*LDMC*				
(Intercept)	**252.99**	**10.42**	**24.29**	**<.001**
HabitatMG	**51.81**	**8.39**	**6.18**	**<.001**
HabitatSDG	**70.44**	**8.57**	**8.22**	**<.001**
speciesCen_jac	**−42.91**	**10.83**	**−3.96**	**<.001**
speciesGal_mol	−19.43	10.39	−1.87	.064
speciesGle_hed	**−38.40**	**13.30**	**−2.89**	**.005**
speciesHer_sph	**−64.62**	**17.03**	**−3.80**	**<.001**
speciesKna_arv	**−66.10**	**10.83**	**−6.10**	**<.001**
speciesLat_pra	**46.00**	**17.03**	**2.70**	**.008**
speciesLot_cor	**−50.23**	**11.13**	**−4.51**	**<.001**
speciesOri_vul	**37.21**	**12.58**	**2.96**	**.004**
speciesPim_sax	−9.73	13.30	−0.73	.466
speciesPla_lan	**−83.52**	**10.59**	**−7.89**	**<.001**
speciesRan_acr	**−41.67**	**12.69**	**−3.28**	**.001**
speciesRan_bul	**−85.20**	**14.12**	**−6.03**	**<.001**
speciesSan_min	25.61	13.19	1.94	.055
speciesSan_off	**83.77**	**14.12**	**5.93**	**<.001**
speciesSec_var	−24.53	15.17	−1.62	.109
*log(SLA)*				
(Intercept)	**2.93**	**0.05**	**54.83**	**<.001**
HabitatMG	**−0.37**	**0.04**	**−8.59**	**<.001**
HabitatSDG	**−0.51**	**0.04**	**−11.55**	**<.001**
speciesCen_jac	**0.11**	**0.06**	**2.06**	**.042**
speciesGal_mol	**0.30**	**0.05**	**5.71**	**<.001**
speciesGle_hed	**0.54**	**0.07**	**7.93**	**<.001**
speciesHer_sph	0.10	0.09	1.14	.256
speciesKna_arv	**0.15**	**0.06**	**2.78**	**.006**
speciesLat_pra	**0.28**	**0.09**	**3.23**	**.002**
speciesLot_cor	**0.40**	**0.06**	**6.96**	**<.001**
speciesOri_vul	**0.13**	**0.06**	**1.99**	**.05**
speciesPim_sax	0.09	0.07	1.35	.179
speciesPla_lan	**0.12**	**0.05**	**2.29**	**.024**
speciesRan_acr	**0.14**	**0.07**	**2.22**	**.029**
speciesRan_bul	**0.39**	**0.07**	**5.41**	**<.001**
speciesSan_min	0.13	0.07	1.90	.06
speciesSan_off	**−0.23**	**0.07**	**−3.20**	**.002**
speciesSec_var	**0.26**	**0.08**	**3.35**	**.001**
*Leaf C*				
(Intercept)	**41.24**	**0.36**	**115.25**	**<.001**
HabitatMG	−0.58	0.30	−1.89	.062
HabitatSDG	0.02	0.31	0.05	.958
speciesCen_jac	**1.23**	**0.36**	**3.44**	**<.001**
speciesGal_mol	0.94	0.90	1.05	.299
speciesGle_hed	**2.09**	**0.47**	**4.43**	**<.001**
speciesHer_sph	**−2.90**	**0.56**	**−5.17**	**<.001**
speciesKna_arv	**2.63**	**0.36**	**7.37**	**<.001**
speciesLat_pra	**3.57**	**0.56**	**6.36**	**<.001**
speciesLot_cor	**2.08**	**0.37**	**5.69**	**<.001**
speciesOri_vul	**3.52**	**0.42**	**8.47**	**<.001**
speciesPim_sax	0.61	0.44	1.39	.169
speciesPla_lan	0.38	0.35	1.10	.275
speciesRan_acr	−0.05	0.42	−0.12	.907
speciesRan_bul	−0.15	0.47	−0.32	.75
speciesSan_min	**1.34**	**0.44**	**3.07**	**.003**
speciesSan_off	0.13	0.47	0.28	.782
speciesSec_var	**1.57**	**0.50**	**3.14**	**.002**
*log(Leaf N)*				
(Intercept)	**0.93**	**0.06**	**15.02**	**<.001**
HabitatMG	**−0.23**	**0.05**	**−4.40**	**<.001**
HabitatSDG	**−0.43**	**0.05**	**−8.15**	**<.001**
speciesCen_jac	**−0.18**	**0.06**	**−2.85**	**.005**
speciesGal_mol	−0.03	0.16	−0.22	.826
speciesGle_hed	−0.09	0.08	−1.15	.253
speciesHer_sph	**−0.22**	**0.10**	**−2.21**	**.03**
speciesKna_arv	−0.05	0.06	−0.83	.409
speciesLat_pra	**0.32**	**0.10**	**3.30**	**.001**
speciesLot_cor	**0.45**	**0.06**	**7.09**	**<.001**
speciesOri_vul	**−0.35**	**0.07**	**−4.89**	**<.001**
speciesPim_sax	**−0.22**	**0.08**	**−2.91**	**.005**
speciesPla_lan	**−0.33**	**0.06**	**−5.50**	**<.001**
speciesRan_acr	**−0.20**	**0.07**	**−2.70**	**.008**
speciesRan_bul	**0.20**	**0.08**	**2.41**	**.018**
speciesSan_min	−0.04	0.08	−0.49	.624
speciesSan_off	−0.12	0.08	−1.50	.136
speciesSec_var	**0.60**	**0.09**	**6.88**	**<.001**
*log(Leaf CN)*				
(Intercept)	**2.79**	**0.06**	**45.63**	**<.001**
HabitatMG	**0.22**	**0.05**	**4.21**	**<.001**
HabitatSDG	**0.43**	**0.05**	**8.30**	**<.001**
speciesCen_jac	**0.21**	**0.06**	**3.39**	**.001**
speciesGal_mol	0.06	0.15	0.38	.708
speciesGle_hed	0.14	0.08	1.80	.076
speciesHer_sph	0.14	0.10	1.48	.141
speciesKna_arv	0.11	0.06	1.87	.065
speciesLat_pra	**−0.24**	**0.10**	**−2.48**	**.015**
speciesLot_cor	**−0.40**	**0.06**	**−6.41**	**<.001**
speciesOri_vul	**0.43**	**0.07**	**6.14**	**<.001**
speciesPim_sax	**0.24**	**0.08**	**3.16**	**.002**
speciesPla_lan	**0.34**	**0.06**	**5.75**	**<.001**
speciesRan_acr	**0.20**	**0.07**	**2.74**	**.007**
speciesRan_bul	**−0.20**	**0.08**	**−2.50**	**.014**
speciesSan_min	0.07	0.07	0.93	.353
speciesSan_off	0.12	0.08	1.57	.12
speciesSec_var	**−0.56**	**0.09**	**−6.55**	**<.001**

*Note*: Presented here are the minimum adequate models for each plant functional trait. For details on the LM, please see the method part. Significant parameters are marked in bold.

Abbreviations: Estim, Estimate; SE, Standard error.

**TABLE A3:** Results of the contrast calculations of the minimum adequate linear models with the R package *emmeans* as described in the methods.

Plant functional trait	Contrast	Estimate	SE	df	*t* Ratio	*p* Value
log(Flower density)	**BG‐MG**	**2.20**	**0.34**	**102.00**	**6.54**	**<.001**
	**BG‐SDG**	**2.73**	**0.34**	**102.00**	**7.96**	**<.001**
	MG‐SDG	0.54	0.24	102.00	2.23	.071
log(Flowers per individual)	**BG‐MG**	**0.70**	**0.14**	**102.00**	**4.99**	**<.001**
	**BG‐SDG**	**0.82**	**0.14**	**102.00**	**5.71**	**<.001**
	MG‐SDG	0.12	0.10	102.00	1.19	.464
log(Plant width)	**BG‐MG**	**0.83**	**0.10**	**103.00**	**8.31**	**<.001**
	**BG‐SDG**	**1.13**	**0.10**	**103.00**	**11.14**	**<.001**
	**MG‐SDG**	**0.31**	**0.07**	**103.00**	**4.13**	**.001**
log(Vegetative mean)	BG‐MG	0.22	0.10	103.00	2.26	.066
	**BG‐SDG**	**0.57**	**0.10**	**103.00**	**5.72**	**<.001**
	**MG‐SDG**	**0.35**	**0.07**	**103.00**	**4.81**	**<.001**
log(Generative mean)	**BG‐MG**	**0.23**	**0.08**	**102.00**	**2.82**	**.016**
	**BG‐SDG**	**0.50**	**0.08**	**102.00**	**5.98**	**<.001**
	**MG‐SDG**	**0.27**	**0.06**	**102.00**	**4.59**	**<.001**
log(Flower size)	BG‐MG	0.09	0.04	102.00	2.22	.073
	**BG‐SDG**	**0.12**	**0.04**	**102.00**	**3.06**	**.008**
	MG‐SDG	0.03	0.03	102.00	1.26	.422
LDMC	**BG‐MG**	**−51.80**	**8.39**	**103.00**	**−6.18**	**<.001**
	**BG‐SDG**	**−70.40**	**8.57**	**103.00**	**−8.22**	**<.001**
	**MG‐SDG**	**−18.60**	**6.25**	**103.00**	**−2.98**	**.010**
log(SLA)	**BG‐MG**	**0.37**	**0.04**	**103.00**	**8.59**	**<.001**
	**BG‐SDG**	**0.51**	**0.04**	**103.00**	**11.55**	**<.001**
	**MG‐SDG**	**0.14**	**0.03**	**103.00**	**4.31**	**.001**
Leaf C	BG‐MG	0.58	0.31	90.00	1.89	.149
	BG‐SDG	−0.02	0.31	90.00	−0.05	.999
	**MG‐SDG**	**−0.59**	**0.23**	**90.00**	**−2.60**	**.029**
log(Leaf N)	**BG‐MG**	**0.23**	**0.05**	**90.00**	**4.40**	**.001**
	**BG‐SDG**	**0.43**	**0.05**	**90.00**	**8.15**	**<.001**
	**MG‐SDG**	**0.20**	**0.04**	**90.00**	**5.03**	**<.001**
log(Leaf CN)	**BG‐MG**	**−0.22**	**0.05**	**90.00**	**−4.21**	**.000**
	**BG‐SDG**	**−0.43**	**0.05**	**90.00**	**−8.30**	**<.001**
	**MG‐SDG**	**−0.21**	**0.04**	**90.00**	**−5.50**	**<.001**

*Note*: Presented are the outputs for the LMs where habitat was a significant parameter and significant differences are marked in bold. For details, see the see Section 2.

Abbreviations: BG, botanical garden; MG, mesophilic grassland; SDG, semi‐dry grassland.

**TABLE A4:** Outputs of the generalised linear models (GLM) across all observed species and habitats.

Phenological stage	Estimate	SE	*z* Value	*p* Value
*FI*_*max*_				
(Intercept)	**4.48**	**0.21**	**21.05**	**<.001**
HabitatMG	**−0.35**	**0.18**	**−2.00**	**.045**
HabitatSDG	**−0.48**	**0.18**	**−2.66**	**.008**
speciesCen_jac	−0.20	0.22	−0.94	.348
speciesGal_mol	−0.19	0.21	−0.89	.375
speciesGle_hed	−0.39	0.26	−1.54	.124
speciesHer_sph	**−1.50**	**0.28**	**−5.31**	**<.001**
speciesKna_arv	−0.43	0.22	−1.94	.052
speciesLat_pra	**−1.61**	**0.28**	**−5.66**	**<.001**
speciesLot_cor	−0.13	0.21	−0.62	.536
speciesOri_vul	−0.12	0.25	−0.49	.628
speciesPim_sax	0.32	0.27	1.20	.229
speciesPla_lan	**−1.60**	**0.22**	**−7.14**	**<.001**
speciesRan_acr	0.22	0.25	0.85	.394
speciesRan_bul	0.31	0.27	1.16	.246
speciesSan_min	−0.42	0.26	−1.63	.104
speciesSan_off	0.08	0.28	0.30	.765
speciesSec_var	−0.59	0.31	−1.88	.060
*FFD*				
(Intercept)	**5.07**	**0.03**	**178.69**	**<.001**
speciesCen_jac	**0.15**	**0.04**	**3.59**	**.001**
speciesGal_mol	−0.02	0.04	−0.38	.707
speciesGle_hed	**−0.51**	**0.06**	**−8.99**	**<.001**
speciesHer_sph	**0.20**	**0.06**	**3.64**	**<.001**
speciesKna_arv	**−0.13**	**0.04**	**−3.00**	**.003**
speciesLat_pra	0.05	0.05	0.92	.359
speciesLot_cor	−0.02	0.04	−0.43	.669
speciesOri_vul	**0.13**	**0.05**	**2.72**	**.006**
speciesPim_sax	**0.25**	**0.05**	**5.15**	**<.001**
speciesPla_lan	−0.05	0.04	−1.24	.215
speciesRan_acr	**−0.31**	**0.05**	**−6.11**	**<.001**
speciesRan_bul	**−0.27**	**0.05**	**−5.12**	**<.001**
speciesSan_min	**−0.22**	**0.05**	**−4.48**	**<.001**
speciesSan_off	**0.25**	**0.05**	**4.95**	**<.001**
speciesSec_var	0.02	0.06	0.39	.696
*FD*_*5*_				
(Intercept)	**4.03**	**0.14**	**28.77**	**<.001**
HabitatMG	**−0.40**	**0.12**	**−3.38**	**<.001**
HabitatSDG	**−0.34**	**0.12**	**−2.78**	**.005**
speciesCen_jac	**−0.34**	**0.14**	**−2.35**	**.019**
speciesGal_mol	**−0.37**	**0.14**	**−2.54**	**.011**
speciesGle_hed	**−0.41**	**0.18**	**−2.30**	**.021**
speciesHer_sph	**−0.61**	**0.21**	**−2.89**	**.004**
speciesKna_arv	−0.13	0.14	−0.91	.364
speciesLat_pra	**−0.82**	**0.22**	**−3.75**	**<.001**
speciesLot_cor	0.04	0.14	0.30	.768
speciesOri_vul	**−0.56**	**0.17**	**−3.27**	**.001**
speciesPim_sax	**0.43**	**0.17**	**2.54**	**.011**
speciesPla_lan	**−1.32**	**0.20**	**−6.61**	**<.001**
speciesRan_acr	0.05	0.17	0.31	.757
speciesRan_bul	**−0.36**	**0.18**	**−1.99**	**.047**
speciesSan_min	**−1.05**	**0.19**	**−5.46**	**<.001**
speciesSan_off	**−0.40**	**0.19**	**−2.08**	**.037**
speciesSec_var	**−0.47**	**0.21**	**−2.26**	**.024**
*LS*_*5*_				
(Intercept)	**5.29**	**0.04**	**150.84**	**<.001**
HabitatMG	**0.12**	**0.03**	**4.33**	**<.001**
HabitatSDG	0.05	0.03	1.83	.067
speciesCen_jac	0.04	0.04	1.03	.301
speciesGal_mol	0.00	0.04	−0.11	.916
speciesGle_hed	**−0.32**	**0.07**	**−4.51**	**<.001**
speciesHer_sph	**−0.16**	**0.05**	**−3.50**	**<.001**
speciesKna_arv	0.07	0.04	1.80	.072
speciesLat_pra	0.01	0.05	0.14	.890
speciesLot_cor	0.03	0.04	0.99	.324
speciesOri_vul	−0.06	0.04	−1.49	.137
speciesPim_sax	0.04	0.04	1.06	.290
speciesPla_lan	−0.03	0.04	−0.71	.479
speciesRan_acr	−0.02	0.04	−0.42	.675
speciesRan_bul	**−0.17**	**0.04**	**−3.93**	**<.001**
speciesSan_min	−0.05	0.04	−1.11	.266
speciesSan_off	**0.11**	**0.05**	**2.37**	**.018**
speciesSec_var	0.02	0.05	0.43	.666
*LS*_*50*_				
(Intercept)	**5.63**	**0.03**	**188.58**	**<.001**
speciesCen_jac	−0.04	0.04	−1.19	.235
speciesGal_mol	−0.08	0.05	−1.64	.100
speciesHer_sph	**−0.23**	**0.04**	**−5.57**	**<.001**
speciesLat_pra	**−0.19**	**0.04**	**−4.35**	**<.001**
speciesLot_cor	**−0.18**	**0.04**	**−4.70**	**<.001**
speciesOri_vul	**−0.10**	**0.04**	**−2.25**	**.024**
speciesPim_sax	0.05	0.07	0.75	.457
speciesPla_lan	**−0.16**	**0.05**	**−3.01**	**.003**
speciesRan_acr	**−0.25**	**0.06**	**−4.43**	**<.001**
speciesRan_bul	**−0.37**	**0.04**	**−8.79**	**<.001**
speciesSan_min	**−0.23**	**0.07**	**−3.10**	**.002**
speciesSan_off	−0.03	0.07	−0.43	.668
speciesSec_var	**−0.22**	**0.05**	**−4.57**	**<.001**
*FRF*				
(Intercept)	**5.32**	**0.02**	**274.01**	**<.001**
speciesCen_jac	0.04	0.03	1.25	.210
speciesGal_mol	**0.06**	**0.03**	**2.33**	**.020**
speciesGle_hed	**−0.46**	**0.04**	**−11.16**	**<.001**
speciesHer_sph	**0.10**	**0.04**	**2.55**	**.011**
speciesKna_arv	**−0.16**	**0.03**	**−5.20**	**<.001**
speciesLat_pra	−0.03	0.05	−0.75	.455
speciesLot_cor	0.04	0.03	1.44	.151
speciesOri_vul	**0.06**	**0.03**	**2.00**	**.045**
speciesPim_sax	**0.19**	**0.03**	**5.72**	**<.001**
speciesPla_lan	−0.05	0.03	−1.74	.082
speciesRan_acr	**−0.26**	**0.04**	**−7.18**	**<.001**
speciesRan_bul	**−0.28**	**0.04**	**−7.31**	**<.001**
speciesSan_min	**−0.19**	**0.04**	**−5.52**	**<.001**
speciesSan_off	**0.16**	**0.03**	**4.70**	**<.001**
speciesSec_var	0.03	0.04	0.65	.514

*Note*: Presented here are the minimum adequate models for each phenological stage. For description of the phenological stages and abbreviations, please see the method part. Significant parameters are marked in bold.

**TABLE A5:** Outputs of the generalised linear models (GLM) across all match species, which occurred in every habitat and across habitats.

Phenological stage	Estimate	SE	*z* Value	*p* Value
*FI*_*max*_ *match*				
(Intercept)	**4.12**	**0.14**	**30.15**	**<.001**
speciesCen_jac	−0.25	0.20	−1.25	.210
speciesGal_mol	−0.23	0.20	−1.18	.240
speciesKna_arv	**−0.47**	**0.20**	**−2.28**	**.023**
speciesLot_cor	−0.21	0.20	−1.05	.294
speciesPla_lan	**−1.62**	**0.21**	**−7.69**	**<.001**
*FFD match*				
(Intercept)	**5.07**	**0.03**	**158.21**	**<.001**
speciesCen_jac	**0.15**	**0.05**	**3.16**	**.002**
speciesGal_mol	−0.02	0.05	−0.33	.739
speciesKna_arv	**−0.13**	**0.05**	**−2.66**	**.008**
speciesLot_cor	−0.02	0.05	−0.38	.705
speciesPla_lan	−0.05	0.05	−1.10	.271
*FD*_*5*_ *match*				
(Intercept)	**3.69**	**0.11**	**33.54**	**<.001**
speciesCen_jac	**−0.33**	**0.17**	**−2.00**	**.045**
speciesGal_mol	**−0.40**	**0.17**	**−2.41**	**.016**
speciesKna_arv	−0.13	0.16	−0.79	.427
speciesLot_cor	0.02	0.16	0.15	.877
speciesPla_lan	**−1.23**	**0.22**	**−5.59**	**<.001**
*LS*_*5*_ *match*				
(Intercept)	**5.34**	**0.04**	**139.67**	**<.001**
HabitatMG	**0.08**	**0.04**	**1.98**	**.048**
HabitatSDG	0.02	0.04	0.49	.624
*LS*_*50*_ *match*				
(Intercept)	**5.63**	**0.04**	**152.58**	**<.001**
speciesCen_jac	−0.04	0.05	−0.96	.335
speciesGal_mol	−0.08	0.06	−1.34	.180
speciesLot_cor	**−0.18**	**0.05**	**−3.84**	**<.001**
speciesPla_lan	**−0.16**	**0.07**	**−2.48**	**.013**
*FRF match*				
(Intercept)	**5.32**	**0.02**	**223.83**	**<.001**
speciesCen_jac	0.04	0.03	1.02	.308
speciesGal_mol	0.06	0.03	1.89	.059
speciesKna_arv	**−0.16**	**0.04**	**−4.31**	**<.001**
speciesLot_cor	0.04	0.03	1.17	.243
speciesPla_lan	−0.05	0.03	−1.43	.154

*Note*: Presented here are the minimum adequate models for each phenological stage. For description of the phenological stages and abbreviations, please see the method part. Significant parameters are marked in bold.

**TABLE A6:** Results of the contrast calculations of the minimum adequate generalised linear models with the R package *emmeans* as described in the methods.

Phenological stage	Contrast	Estimate	SE	df	*z* Value	*p* Value
FI_max_ (all species)	BG‐MG	0.35	0.18	Inf	2.00	.112
	**BG‐SDG**	**0.48**	**0.18**	**Inf**	**2.66**	**.021**
	MG‐SDG	0.13	0.13	Inf	1.03	.561
FD_5_ (all species)	**BG‐MG**	**0.40**	**0.12**	**Inf**	**3.38**	**.002**
	**BG‐SDG**	**0.34**	**0.12**	**Inf**	**2.78**	**.015**
	MG‐SDG	−0.06	0.09	Inf	−0.71	.760
LS_5_ (all species)	**BG‐MG**	**−0.12**	**0.03**	**Inf**	**−4.33**	**<.001**
	BG‐SDG	−0.05	0.03	Inf	−1.83	.159
	**MG‐SDG**	**0.07**	**0.02**	**Inf**	**3.32**	**.003**
LS_5_ (match species)	BG‐MG	−0.08	0.04	Inf	−1.98	.118
	BG‐SDG	−0.02	0.04	Inf	−0.49	.876
	**MG‐SDG**	**0.06**	**0.02**	**Inf**	**2.79**	**.015**

*Note*: Presented are the outputs for the GLMs where habitat was a significant parameter, results are given on the log scale and significant differences are marked in bold. For description of the phenological stages, please see the method part.

Abbreviations: BG, botanical garden; MG, mesophilic grassland; SDG, semi‐dry grassland.
