# Supplementary material for: Habitat conditions filter stronger for functional traits than for phenology in herbaceous species
Source: Ecol Evol. 2024 Jun 4;14(6):e11505. doi: 10.1002/ece3.11505 (PMC11148399; doi:10.1002/ece3.11505)
Supplement: Supplementary file 1 — Data S1. [file ECE3-14-e11505-s001.docx]

# Supporting Information on the manuscript:

# Title

Habitat conditions filter stronger for functional traits than for phenology in herbaceous species

# Author details

Till J. Deilmann^1^, Josephine Ulrich^1,2^ and Christine Römermann^1,2^

ORCIDs: Till J. Deilmann <https://orcid.org/0000-0002-9521-1825>, Josephine Ulrich <https://orcid.org/0000-0002-5873-8804>, Christine Römermann <https://orcid.org/0000-0003-3471-0951>

^1^Institute of Ecology and Evolution, Friedrich-Schiller-University Jena, Philosophenweg 16, 07743 Jena, Germany

^2^German Centre for Integrative Biodiversity Research (iDiv) Halle-Jena-Leipzig

*Author for correspondence:*

*Till J. Deilmann*

*Email:* [*till.jonathan.deilmann@uni-jena.de*](mailto:till.jonathan.deilmann@uni-jena.de)

# Supplementary Figures – Partial dependency plots


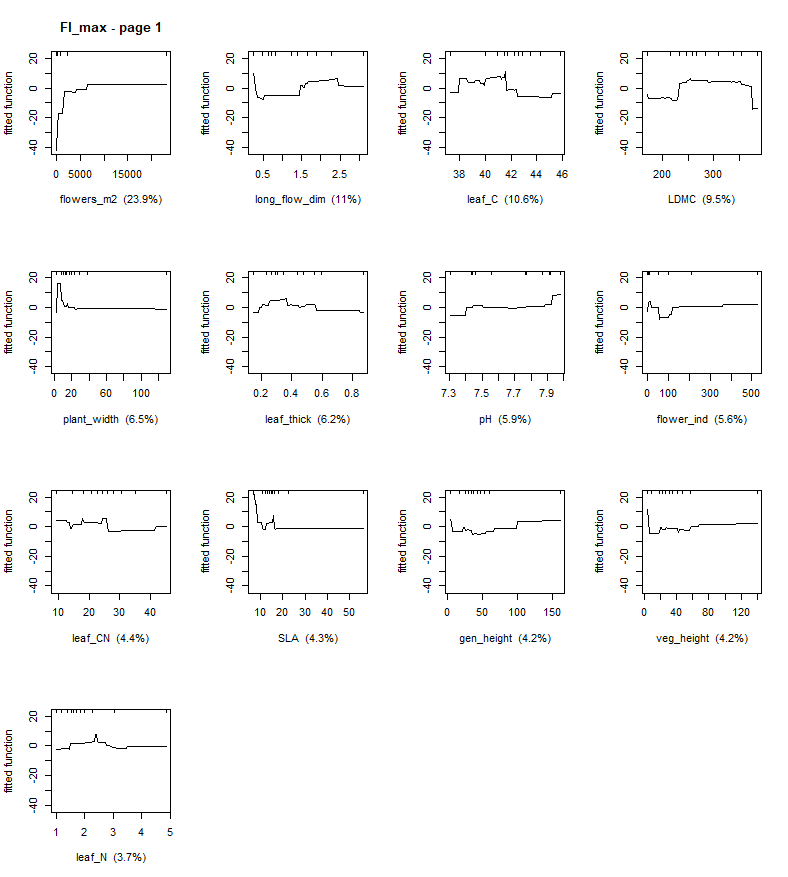

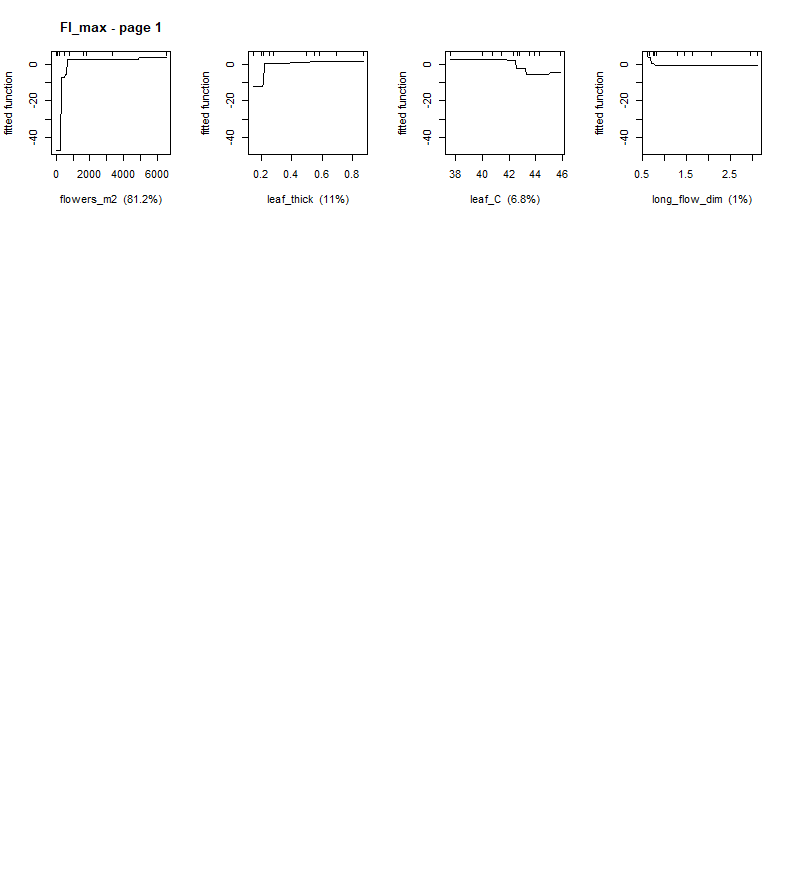


FI_max_ - All

FI_max_ - BG

Figure S1: Partial dependence plots of the boosted regression trees for maximum flower intensity (FI_max_) for all habitats together and for each habitat separately, showing the relative change of FI_max_ integrating over each independent parameter. BG = Botanical Garden, MG = mesophilic grassland, SDG = semi-dry grassland.


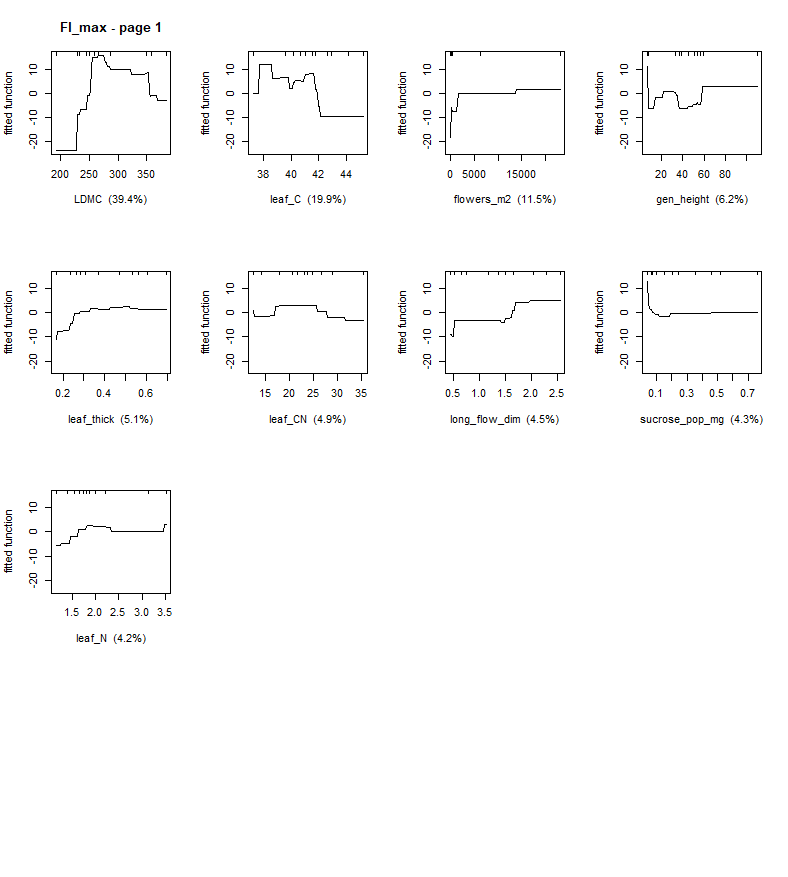

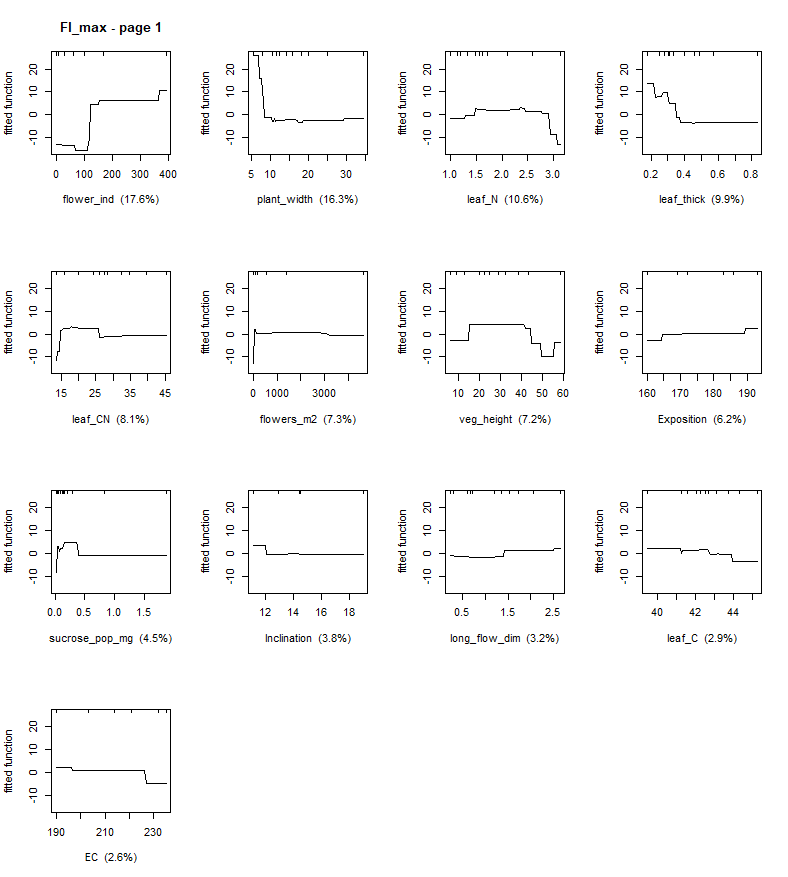


FI_max_ - SDG

FI_max_ - MG

Continuation of Figure S1.


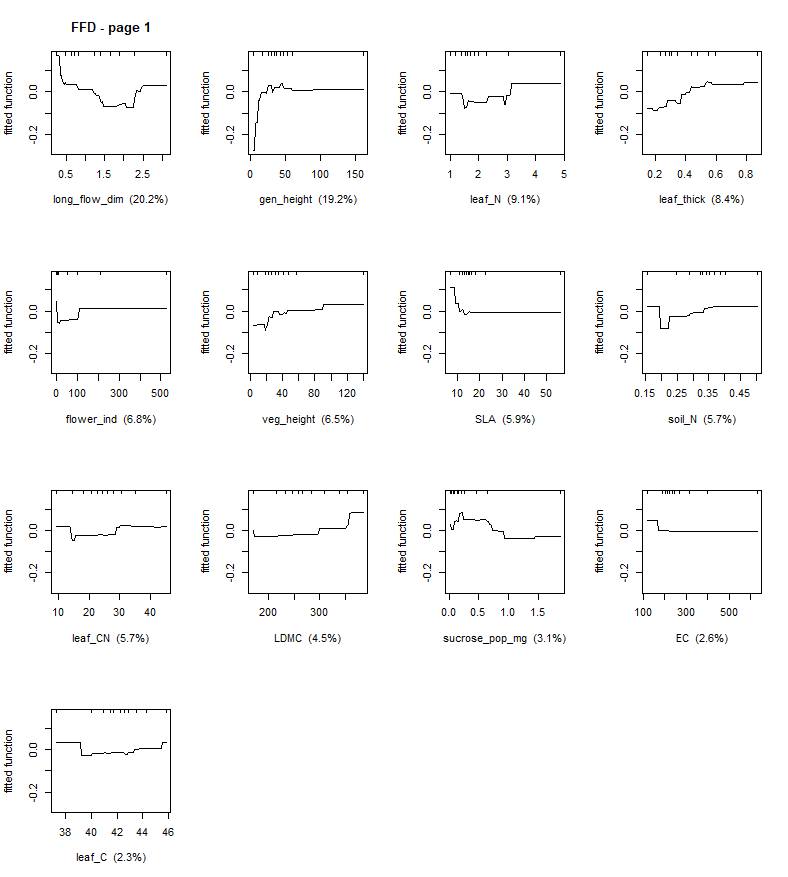

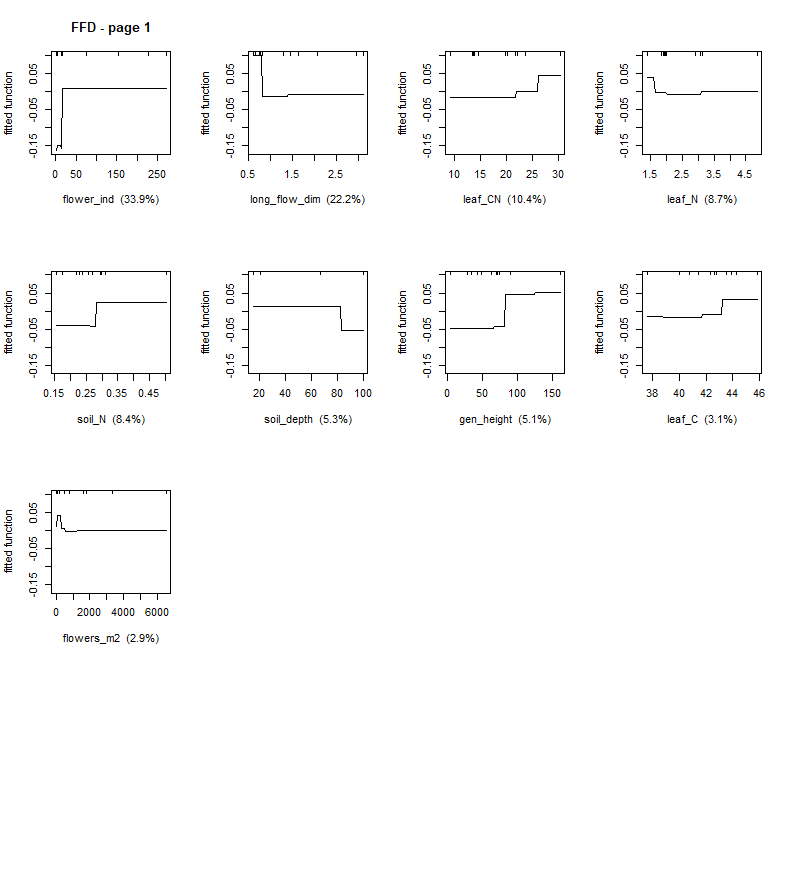


FFD - All

FFD - BG

Figure S2: Partial dependence plots derived from the boosted regression trees for first flowering day (FFD) for all habitats together and for each habitat separately, showing the relative change of FFD integrating over each independent parameter. BG = Botanical Garden, MG = mesophilic grassland, SDG = semi-dry grassland.

Continuation of Figure S2.


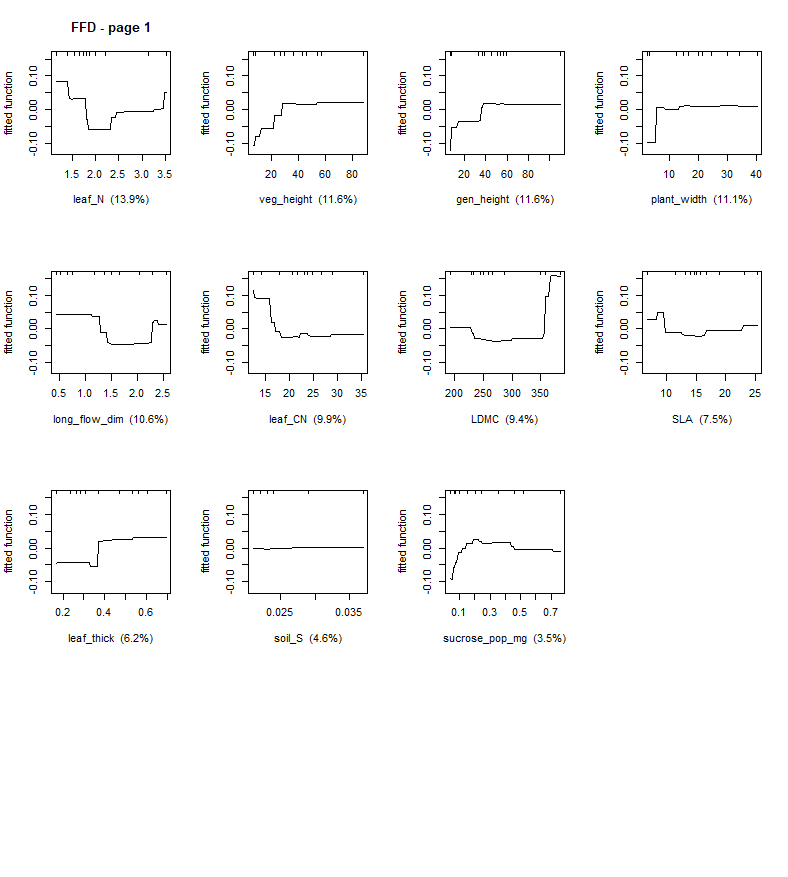

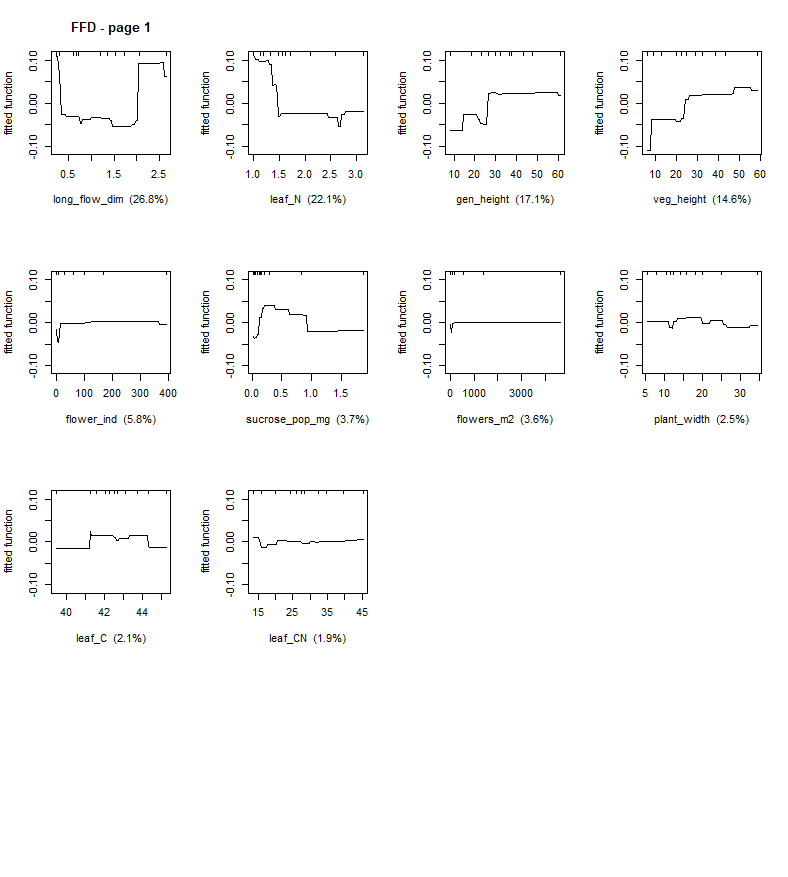


FFD - SDG

FFD - MG


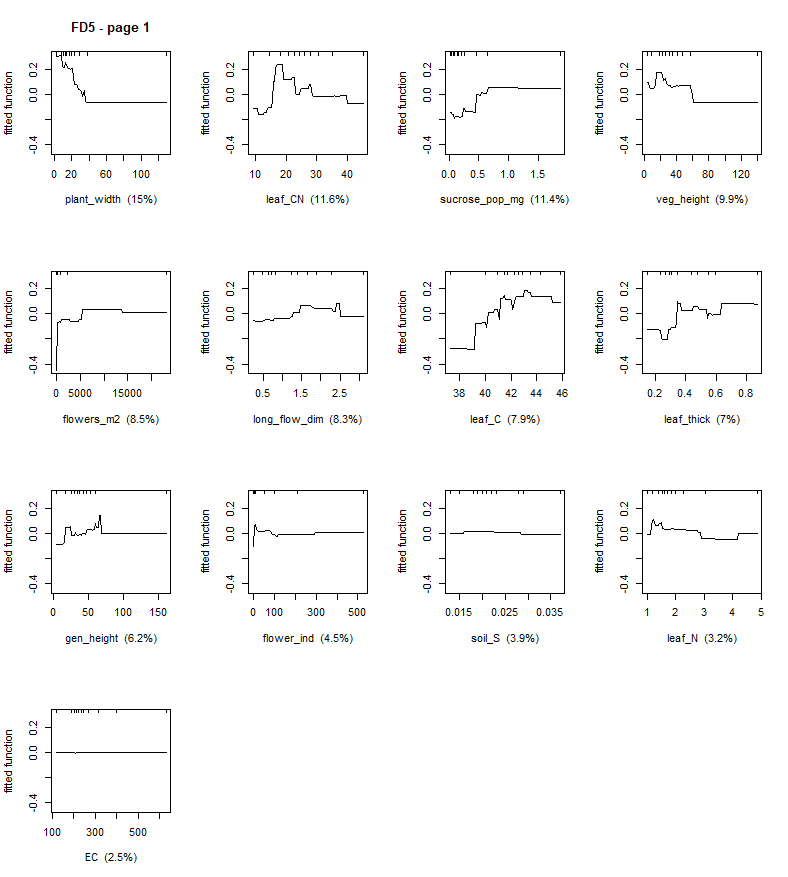

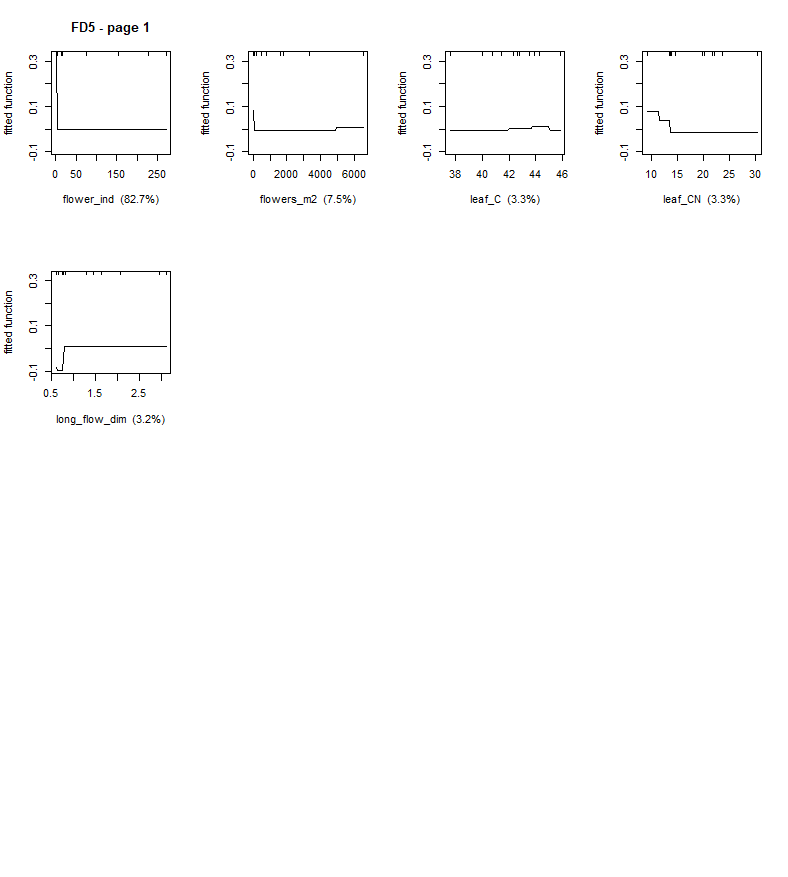


FD_5_ – All

FD_5_ - BG

Figure S3: Partial dependence plots derived from the boosted regression trees for flowering duration with at least 5% opened flowers (FD_5_) for all habitats together and for each habitat separately, showing the relative change of FD_5_ integrating over each independent parameter. BG = Botanical Garden, MG = mesophilic grassland, SDG = semi-dry grassland.

Continuation of Figure S3.


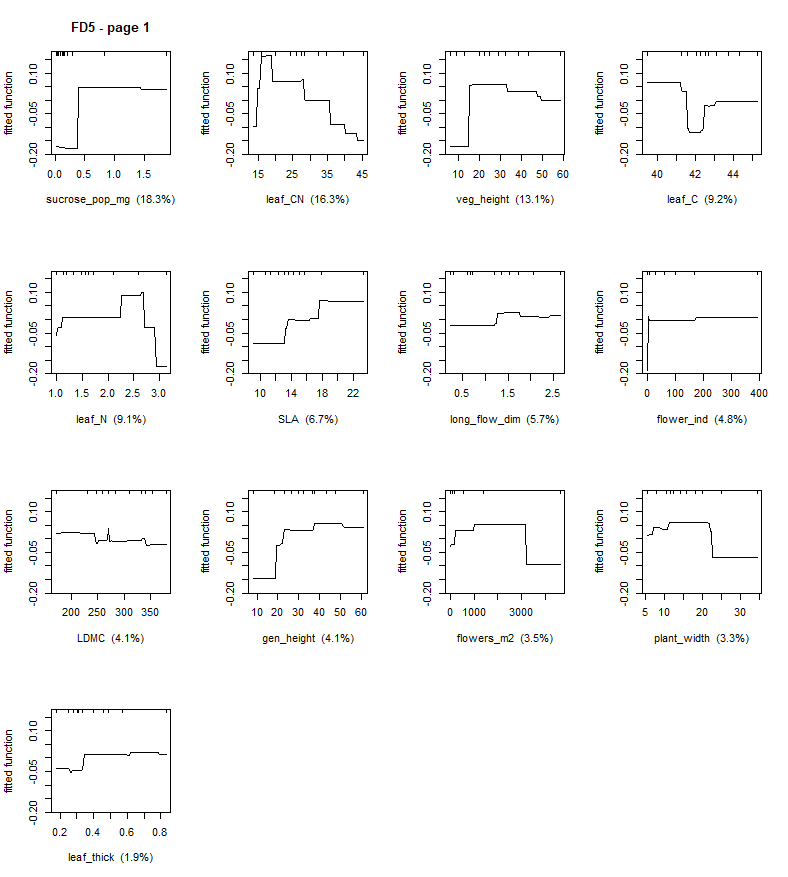

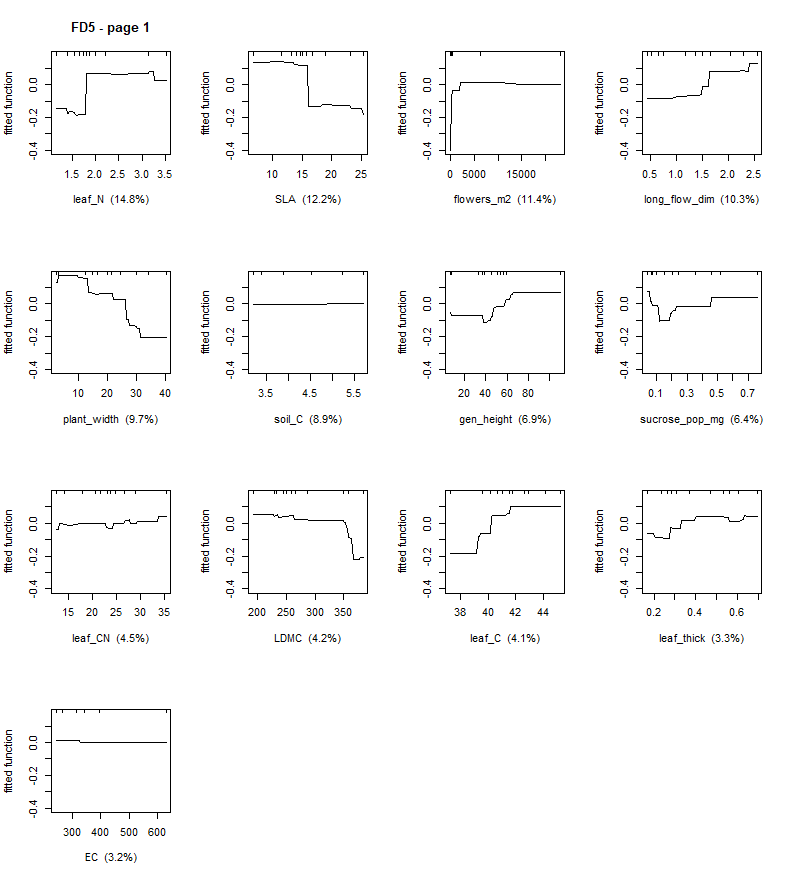


FD_5_ - SDG

FD_5_ - MG

Figure S4: Partial dependence plots derived from the boosted regression trees for the day reaching 5% leaf senescence (LS_5_) for all habitats together and for each habitat separately, showing the relative change of LS_5_ integrating over each independent parameter. BG = Botanical Garden, MG = mesophilic grassland, SDG = semi-dry grassland.


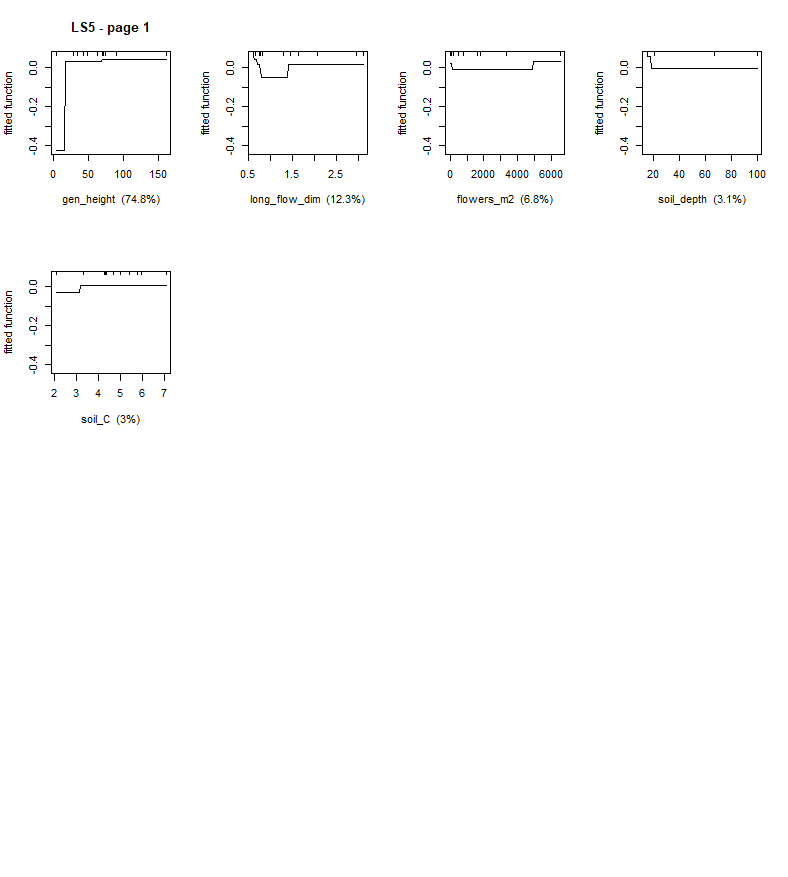

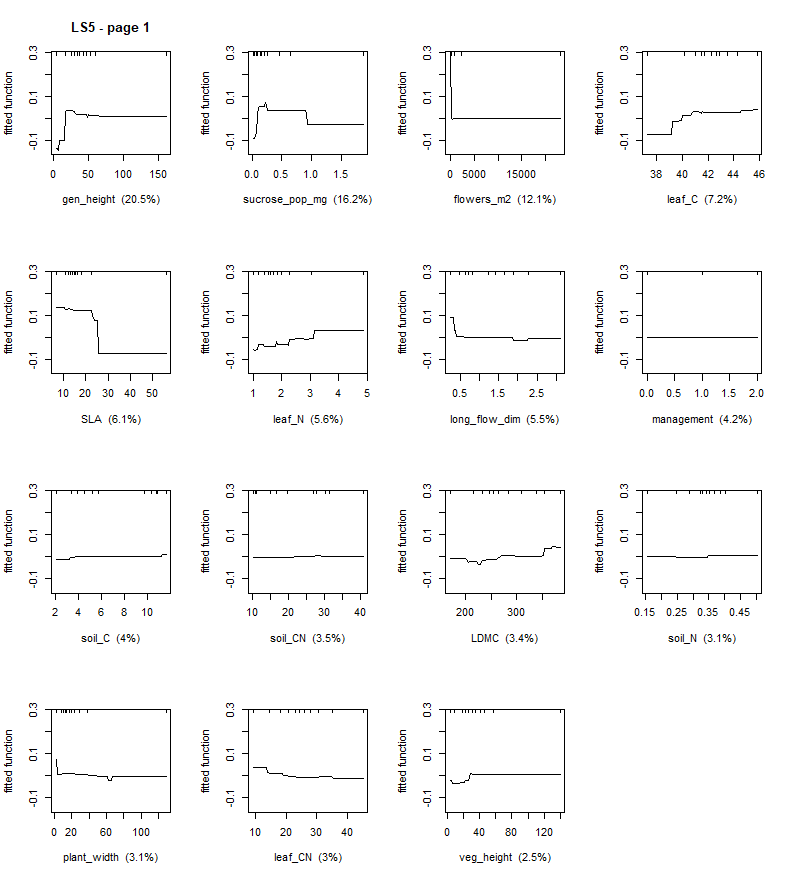


LS_5_ - All

LS_5_ - BG


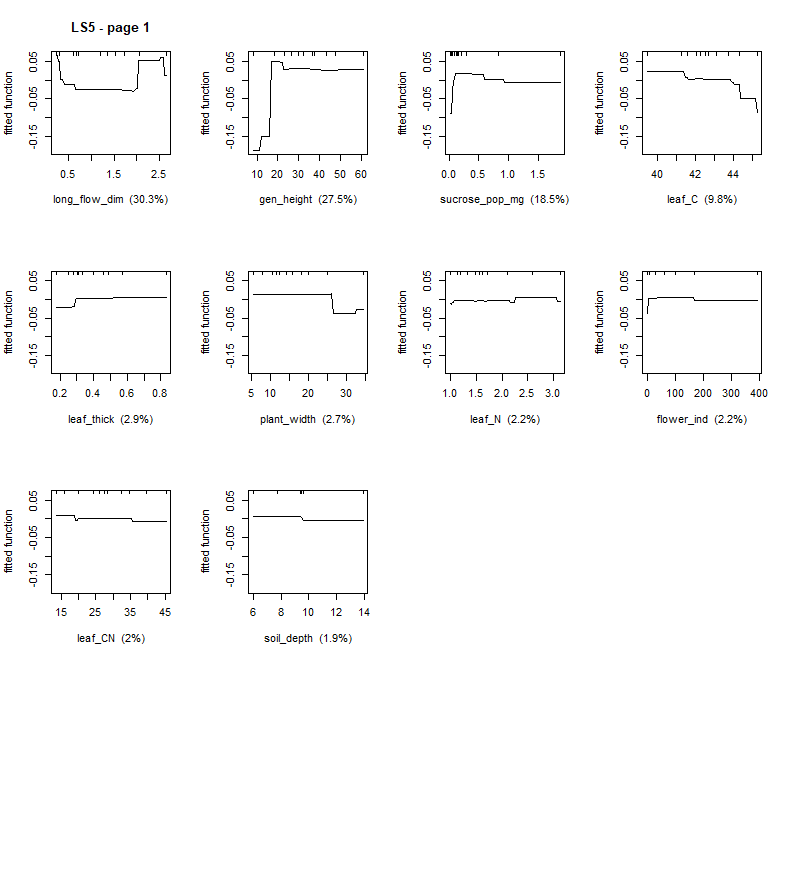

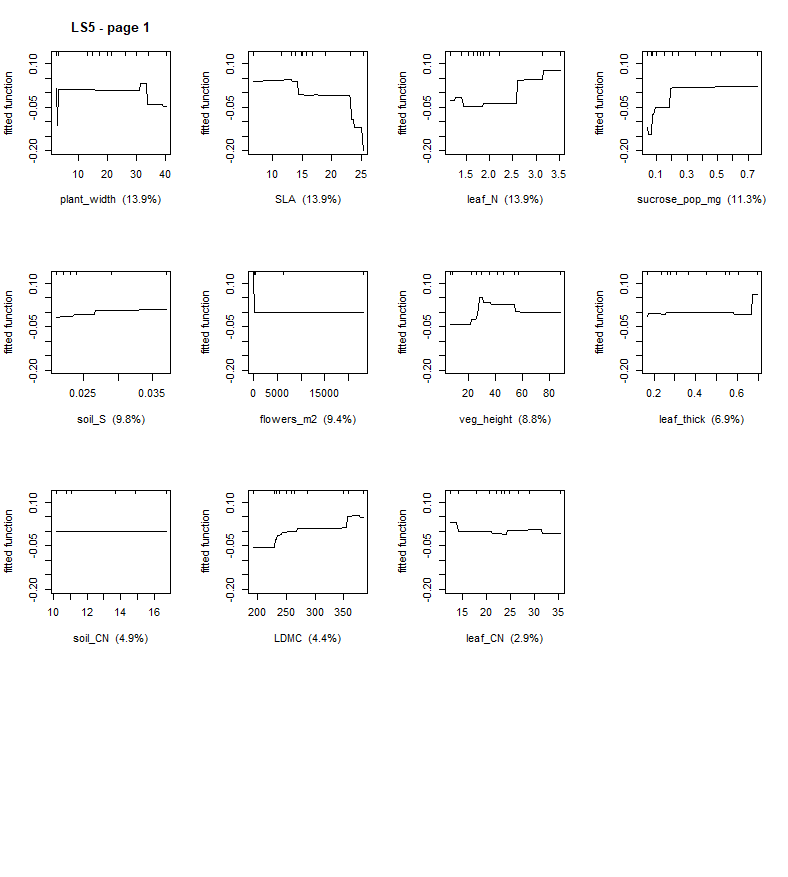


LS_5_ - SDG

LS_5_ - MG

Continuation of Figure S4.

Figure S5: Partial dependence plots derived from the boosted regression trees for the day reaching 50% leaf senescence (LS_50_) for all habitats together and for each habitat separately, showing the relative change of LS_50_ integrating over each independent parameter. BG = Botanical Garden, MG = mesophilic grassland, SDG = semi-dry grassland.


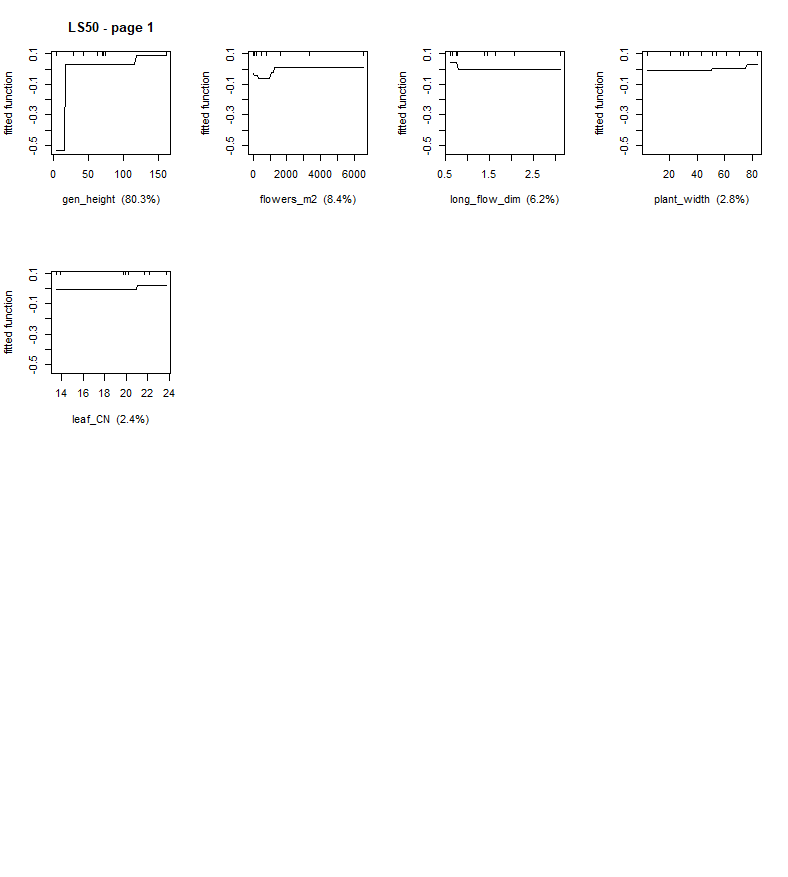

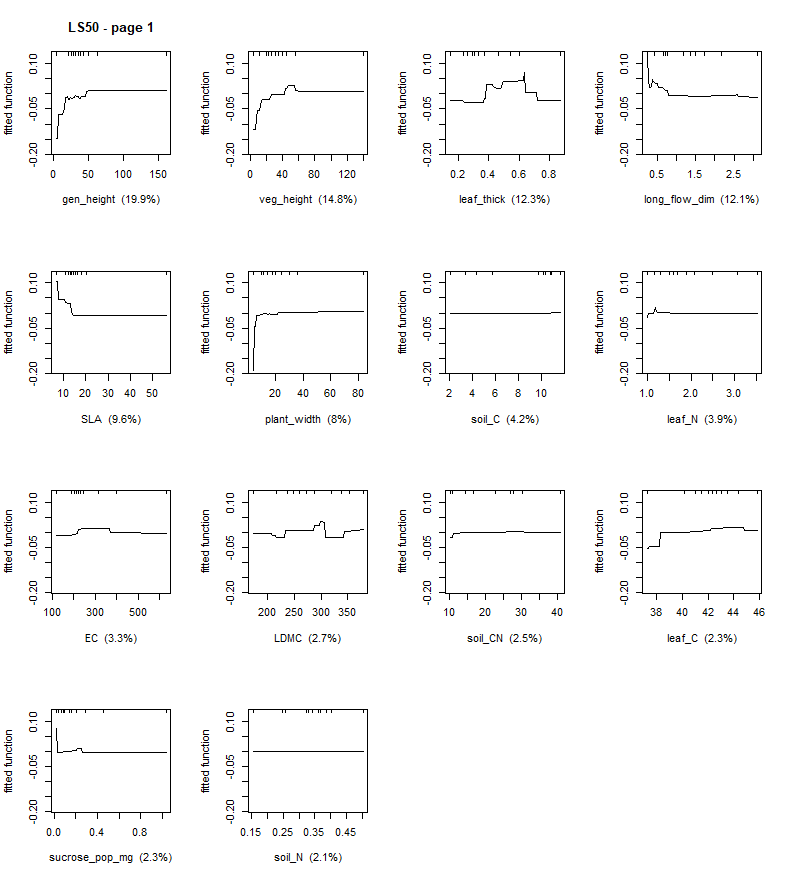


LS_5_ - All

LS_5_ - BG

Continuation of Figure S5.


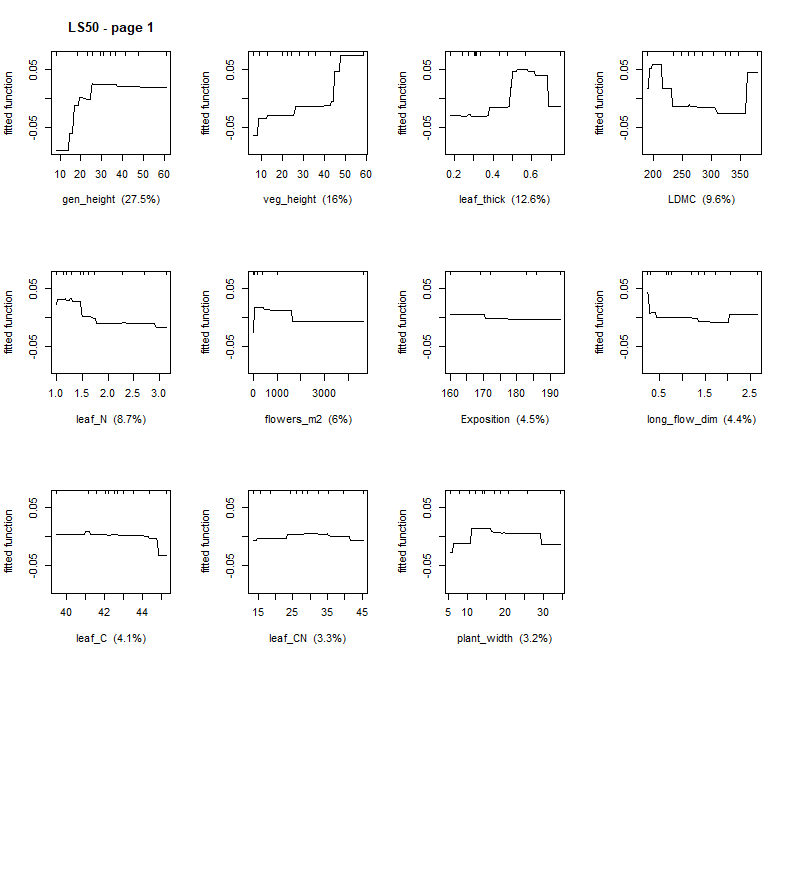

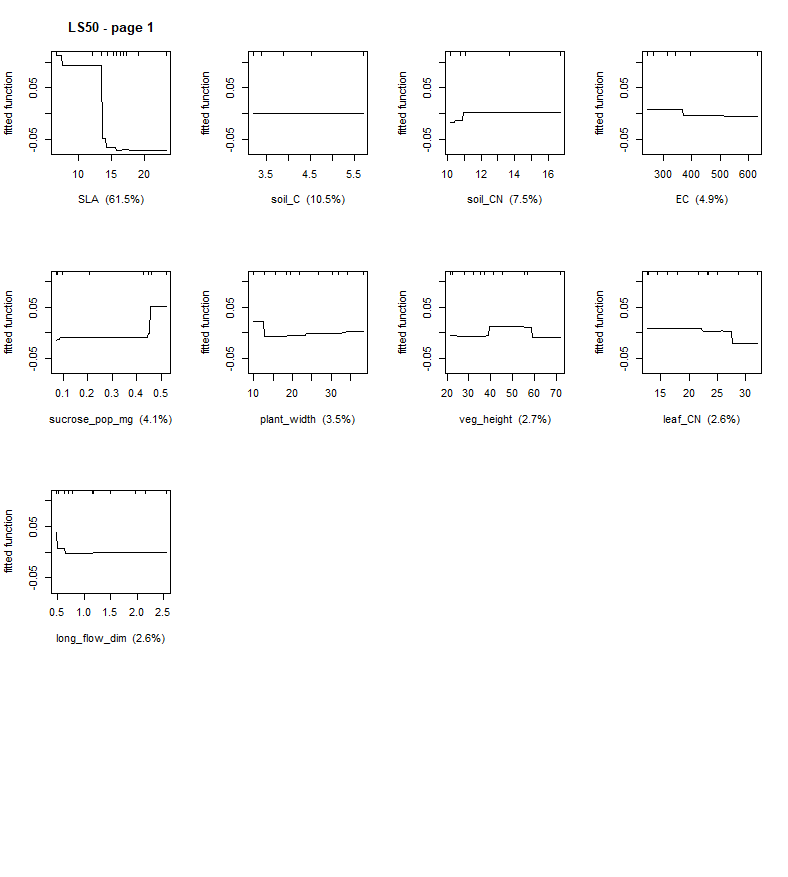


LS_5_ - SDG

LS_5_ - MG

Figure S6: Partial dependence plots derived from the boosted regression trees for the day of the first ripe fruit (FRF) for all habitats together and for each habitat separately, showing the relative change of FRF integrating over each independent parameter. BG = Botanical Garden, MG = mesophilic grassland, SDG = semi-dry grassland.


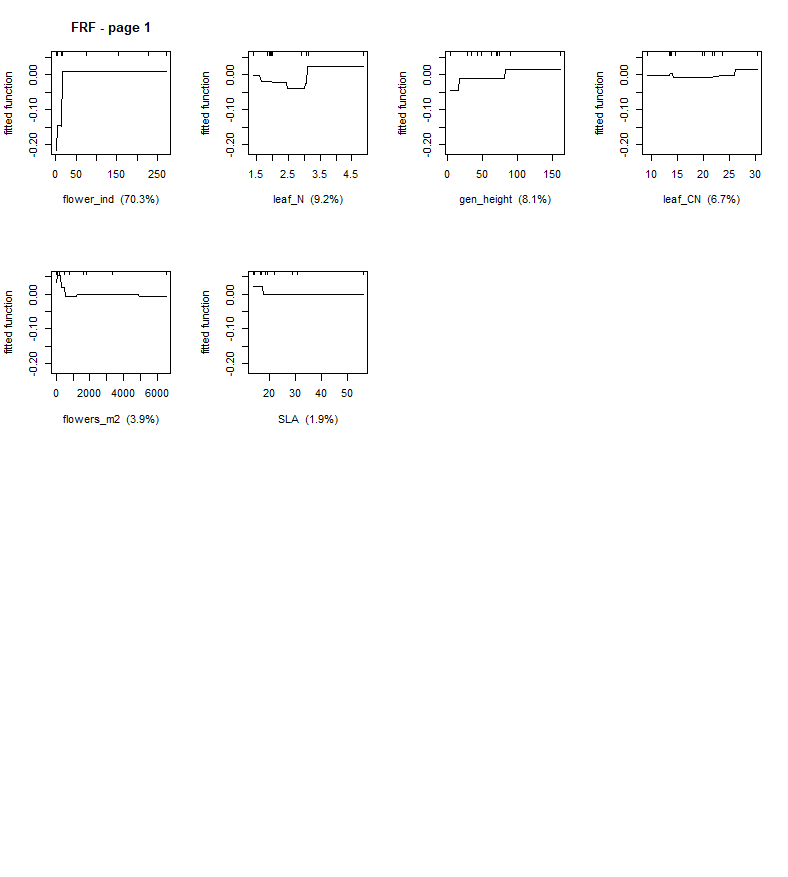

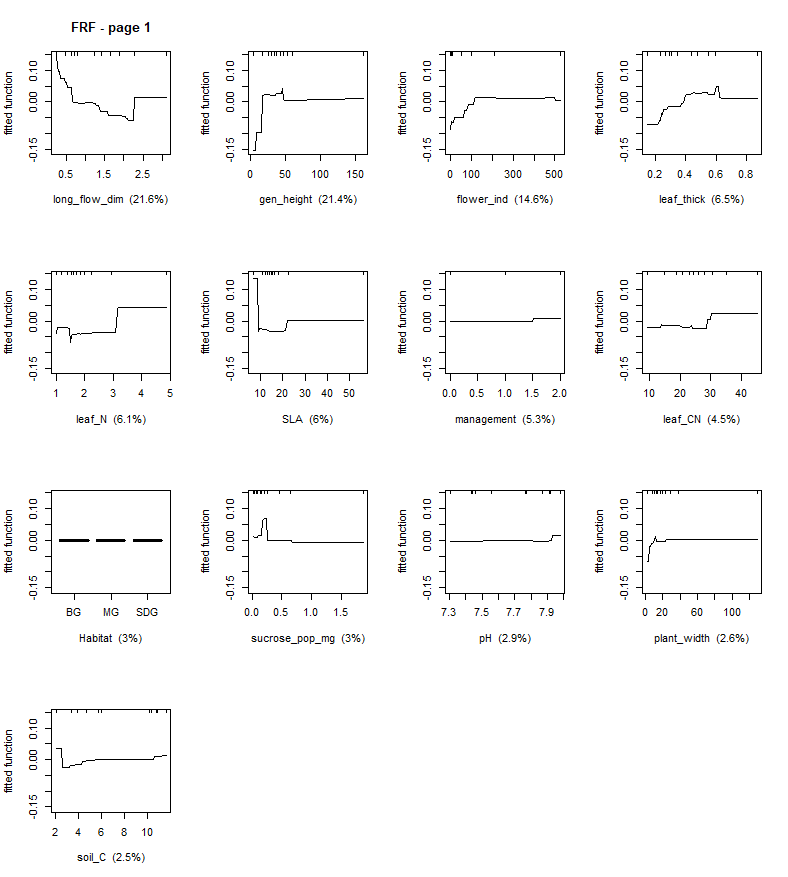


FRF - All

FRF - BG

Continuation of Figure S6.


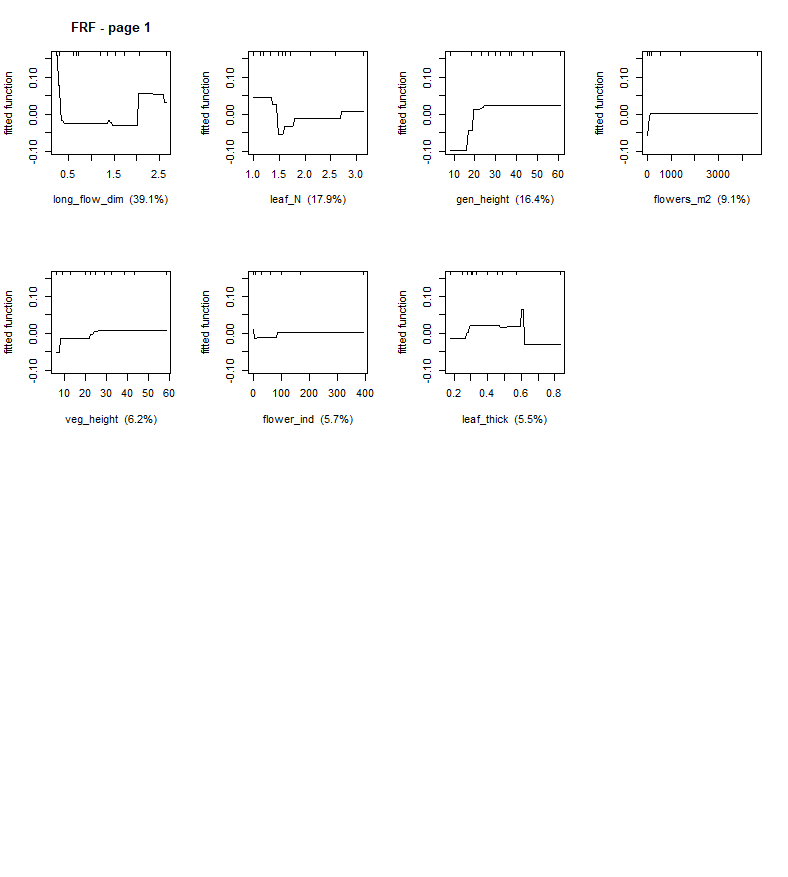

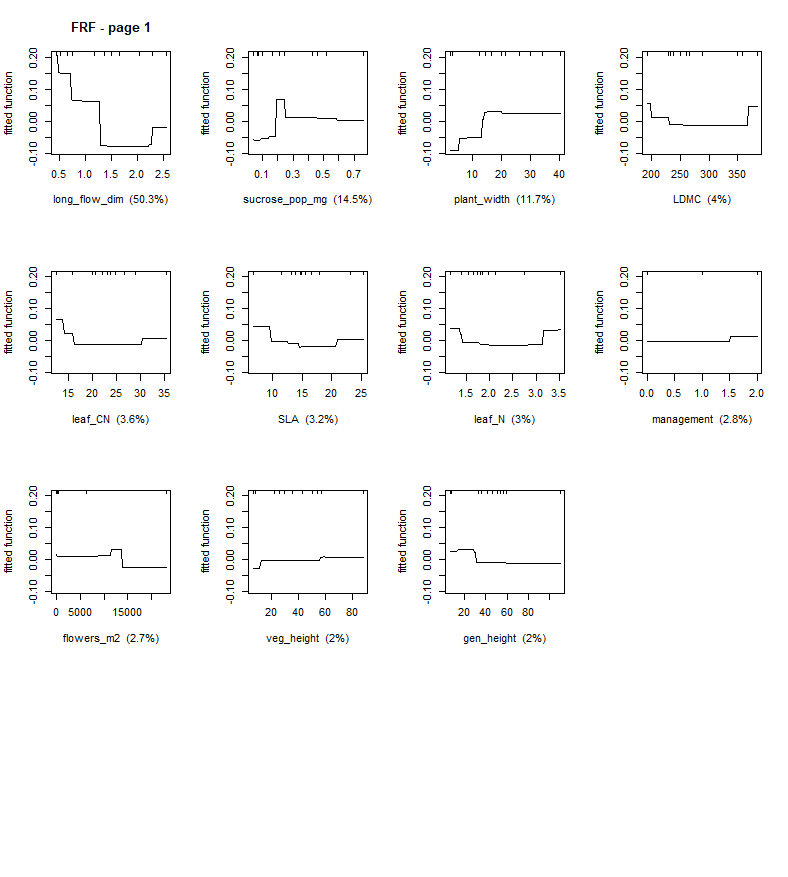


FRF - SDG

FRF - MG
